# Shared CD4^+^ T cell receptor specificity groups in Crohn’s disease and ulcerative colitis

**DOI:** 10.1172/jci.insight.195354

**Published:** 2026-06-09

**Authors:** Joshua E. Chan, Azam Mohsin, Jens Krijgsman, Ciska Lindelauf, Qinghui Mu, Brianna Cavalla, Xuhuai Ji, Sarah E. Streett, Vincent van Unen, Mark M. Davis

**Affiliations:** 1 Institute of Immunity, Transplantation and Infection, and; 2 Department of Microbiology and Immunology, Stanford University School of Medicine, Stanford, California, USA.; 3 Department of Immunology, Leiden University Medical Center, Leiden, Zuid-Holland, Netherlands.; 4 Division of Hematology, Department of Medicine,; 5 Department of Gastroenterology & Hepatology, Stanford University School of Medicine, Stanford, California, USA.; 6 Human Immune Monitoring Center, and; 7 Howard Hughes Medical Institute, Stanford University School of Medicine, Stanford, California, USA.

**Keywords:** Gastroenterology, Immunology, Adaptive immunity, Bioinformatics

## Abstract

Inflammatory bowel disease (IBD), encompassing ulcerative colitis (UC) and Crohn’s disease (CD), is marked by chronic intestinal inflammation and dysregulated immunity. Although UC and CD affect different areas of the gastrointestinal tract, both diseases share aberrant CD4^+^ memory T cell responses, with HLA-DRB1 as a major genetic risk factor. HLA-DRB1 encodes MHC class II molecules that influence the CD4^+^ T cell receptor (TCR) repertoire, yet how these genotypes shape TCR specificity in IBD remains unclear. Here, we genotyped HLA-DRB1 and profiled 3.13 million TCRβ sequences from circulating memory CD4^+^ T cells in 33 IBD patients (20 UC, 13 CD) and 14 healthy controls. Using the GLIPH2 algorithm, we distilled 468,441 candidates based on CDR3 amino acid motifs into 440 high-confidence TCR specificity groups significantly enriched among individuals sharing HLA-DRB1 alleles. Notably, 5 specificity groups were IBD-enriched and were shared between UC and CD, suggesting common antigen targets in both diseases. We also observed increased frequencies of clonally expanded cytotoxic *GZMB^+^PRF1^+^* memory CD4^+^ T cells and KIR^+^CD8^+^ T cells in a subset of risk-allele carriers with IBD. These findings elucidate distinct, HLA-linked TCR specificity groups in IBD and provide mechanistic insights that may advance antigen discovery and personalized medicine.

## Abstract 2

Crohn's disease and ulcerative colitis patients share patterns in their immune cells, suggesting both conditions may be driven by some of the same unknown triggers.

## Graphical abstract

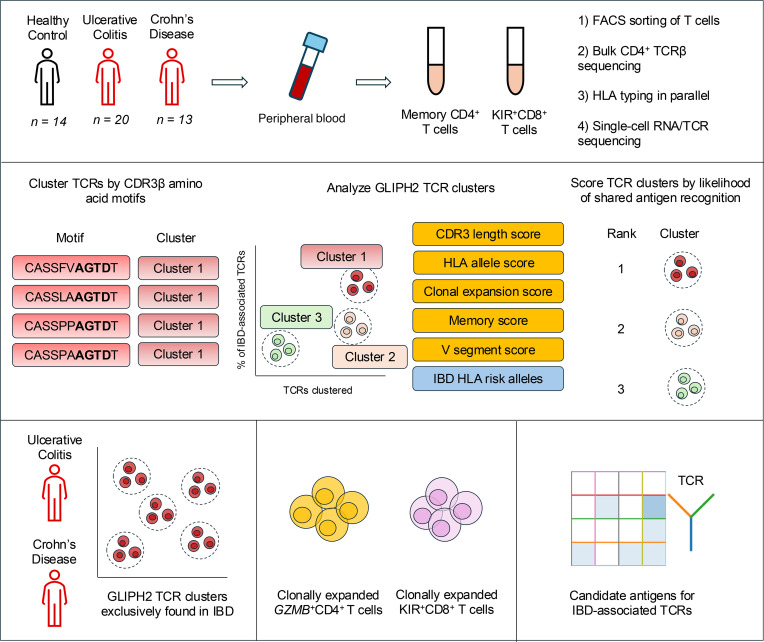

## Introduction

Inflammatory bowel disease (IBD) encompasses a spectrum of chronic gastrointestinal disorders affecting approximately 1.3% of adults in the United States (1). Despite its increasing incidence, the exact causes of IBD remain elusive, and there is no definitive cure. IBD manifests primarily in two major forms: Crohn’s disease (CD) and ulcerative colitis (UC), both characterized by relapsing symptoms like chronic diarrhea, abdominal pain, rectal bleeding, and fatigue (2). Current treatments, typically lifelong and expensive pharmacotherapy (3, 4), often fail to provide consistent relief and have unpredictable therapeutic responses (5). Furthermore, these options achieve clinical remission only in 30%–40% of patients, and the rates of surgical resection for refractory IBD remain substantial. Therefore, there is an urgent need for an improved understanding of this disease and the development of more effective therapies.

The pathogenesis of IBD is multifactorial (6), encompassing immunological factors like aberrant CD4^+^ T cell responses within the intestine (7), environmental factors like intestinal microbiota composition (8), and genetic determinants, notably the human leukocyte antigen (HLA) locus (9, 10). Among the HLA alleles, the HLA-DRB1 locus emerges as a significant genetic risk factor for both CD and UC (10), especially HLA-DRB1*01:03. For pediatric-onset IBD, this represents a substantial portion of the genetic predisposition (11). Moreover, since variation in HLA allotypes dictates antigen binding and the shaping of the T cell receptor (TCR) repertoire (12), certain HLA alleles likely influence the selection of specific T cell populations in IBD.

Despite the scientific consensus that specific HLA-DRB1 alleles are an immunogenetic risk in IBD and that CD4^+^ T cells contribute to the disease’s pathogenesis, an integrated analysis of these components remains to be conducted. Earlier studies suggest the presence of rare, disease-associated CD4^+^ T cells in the peripheral blood of IBD patients (13–15). These observations suggest that interactions between specific HLA-II proteins and TCRs, key for orchestrating CD4^+^ T cell responses, might be instrumental in IBD pathogenesis. Therefore, we hypothesized that distinct CD4^+^ T cell subsets express TCRs that recognize antigens associated with IBD, thereby driving the underlying intestinal inflammation in patients with high-risk HLA class II alleles.

Here, we compared the memory CD4^+^ T cell phenotypes and repertoires between HLA-typed IBD patients and healthy controls, employing flow cytometry, bulk TCRβ sequencing, and single-cell TCR sequencing coupled with transcriptomic analysis. The GLIPH (Grouping of Lymphocyte Interactions by Paratope Hotspots) family of algorithms enables the rapid clustering of TCRs according to the similarities of their CDR3 sequences, as those clustering together have a high probability of sharing the same peptide-MHC specificity (16). Here we used the GLIPH2 variant, which can cluster millions of TCRs from TCR sequencing data (17). We were able to discover 5 memory CD4^+^ TCR specificity groups uniquely prevalent in IBD patients and 10 groups predominant in healthy individuals. These IBD-associated groups varied by disease subtypes and states of disease activity, but a notable finding here was that all 5 of the TCR groups identified were shared between the CD and UC patients, indicating that several target antigens are shared, even though many aspects of these diseases are distinct. The risk allele HLA-DRB1*12:01 was the predominant allele in 2 of the 5 IBD-enriched groups. Additionally, a subset of IBD patients with high-risk HLA alleles exhibited an increase in clonally expanded cytotoxic *GZMB^+^PRF1^+^* memory CD4^+^ T cells and KIR^+^CD8^+^ T cells in peripheral blood, suggesting that these genetic and cellular phenotypes could be a major driver of pathogenesis.

## Results

### Investigating the influence of HLA-DRB1 alleles on IBD risk.

The cohort analyzed here included 14 healthy controls and 33 IBD patients — 20 with UC and 13 with CD — detailed in Table 1. Recognizing the significant role of the HLA-DRB1 region as a genetic risk factor in IBD (10), which affects both susceptibility and the progression of the disease, we stratified patients by their HLA-DRB1 allele composition. This stratification incorporated significant IBD-associated HLA-DRB1 allotypes, identified by Goyette et al. (9), based on extensive HLA region SNP typing in a large IBD cohort.

In our analysis, the risk allele DRB1*11:01 was the most frequent among UC patients at 12.5% (Supplemental Table 1; supplemental material available online with this article; https://doi.org/10.1172/jci.insight.195354DS1). Interestingly, the risk allele DRB1*12:01 was found in 10% of UC patients and 11.5% of CD patients but was absent in the healthy control group. Conversely, the risk allele DRB1*15:01 was common among both healthy individuals and CD patients.

**Figure 1:**
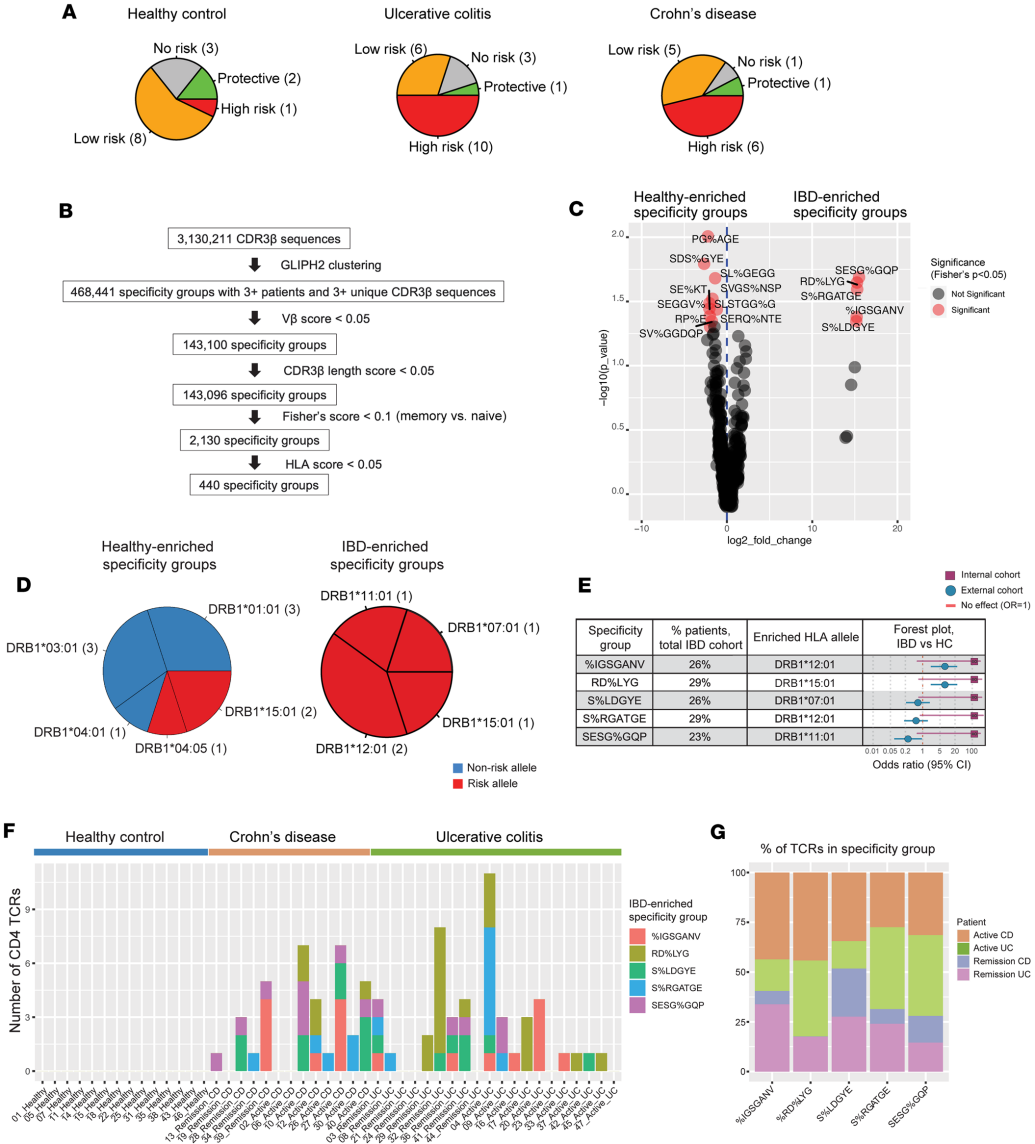
GLIPH2 to prioritize IBD-associated TCR specificity groups. (**A**) Pie charts illustrating the distribution of HLA-DRB1 alleles per risk group in the cohort (healthy controls, *n* = 14; UC, *n* = 20; and CD, *n* = 13). (**B**) GLIPH2 filtering workflow of our cohort’s TCRs, yielding 5 significantly IBD-enriched and 10 significantly healthy-enriched specificity groups. Memory repertoire enrichment score was determined with a Fisher’s test against reference naive CD4^+^ T cell repertoire datasets. (**C**) Four hundred forty specificity groups were obtained after standard GLIPH2 filtering; those significantly enriched in either healthy controls or IBD patients are shown in red. On the left side of the graph (log_2_ fold change < 0) are the specificity groups enriched in TCRs from healthy controls, and on the right (log_2_ fold change > 0) are specificity groups enriched in TCRs from IBD patients. (**D**) Dominant HLA-II allele in healthy-enriched and IBD-enriched TCR specificity groups. In parentheses are the numbers of patients who bear each HLA-II allele. (**E**) Enrichment of our study’s 5 IBD-enriched specificity groups, in an external, European dataset of 32 IBD patients and 24 healthy controls. (**F**) Distribution of memory CD4^+^ TCRs, stratified by disease state (remission vs. active) and subtype (CD vs. UC), that constitute each IBD-enriched specificity group. (**G**) Percentage of TCRs in active and remission CD and UC specificity groups.

To further assess the distribution of specific HLA-DRB1 alleles, we categorized participants based on their composition of IBD risk alleles (9). Categories included “high risk” for individuals with 2 risk alleles, “low risk” for those with 1, “no risk” for individuals without risk alleles, and “protective” for those with at least 1 protective allele. Our findings revealed that only 7.1% of healthy controls carried high-risk alleles, whereas 50% of UC patients (χ^2^ test, *P* = 0.024) and 46% of CD patients (*P* = 0.061) harbored high-risk alleles (Figure 1A). Thus, these findings suggest a relatively higher prevalence of high-risk HLA-DRB1 alleles among IBD patients in our study cohort compared with healthy counterparts.

### Assessing the T cell repertoire in relation to disease characteristics.

To compare the TCR repertoires among HLA-typed patients and healthy controls, we conducted deep sequencing of the TCRβ chains from CD45RA^–^CD3^+^CD4^+^ memory T cells, sorted by flow cytometry. This analysis resulted in a collective dataset of 3,130,211 TCRβ sequences, of which 1,936,565 sequences represented unique TCR clonotypes.

We then explored whether there was a link between the diversity of the TCR repertoire and various aspects of the disease, such as disease status (remission and active disease), subtype (CD, UC), or degree of HLA-DRB1 risk. Our analysis showed no significant change in the number of T cell clones, unique T cell clonotypes, and repertoire diversity in IBD (Supplemental Figure 1, A–C). We did note greater clonotype sharing (~2,000 unique clonotypes) among several CD patients compared with clonotype sharing among healthy controls (Supplemental Figure 1D). Overall, however, the diversity and expansion of the T cell repertoire were not directly correlated with the activity or type of IBD.

### Exploring TCR specificity groups and their association with HLA-DRB1 risk alleles in IBD.

Without a clear link between disease phenotype and individual TCR clonotypes, we shifted our analysis to clustering TCRs into specificity groups based on their CDR3 amino acid motifs and predicted binding to the same HLA-II–restricted antigens. Using GLIPH2 (16, 17), a CDR3 motif–finding algorithm, we analyzed 3,130,211 TCRβ sequences from the entire cohort and identified 468,441 candidate specificity groups. We then applied sequential significance filters: TCRβ V-segment usage bias (cluster-level enrichment of V-gene usage vs. background) retaining 143,100/468,441 (30.55%); CDR3 length bias (cluster not explained by length alone) retaining 143,096/143,100 (99.97%); memory enrichment (memory vs. naive T cell reference) retaining 2,130/143,096 (1.49%); and shared HLA-DRB1 association across donors retaining 440/2,130 (20.66%). The largest attrition occurred at the memory-enrichment step, thereby preferentially retaining clusters whose shared CDR3 features are overrepresented in memory T cells, consistent with antigen-driven selection. In total, the analysis yielded 440 high-confidence specificity groups (Figure 1B).

Of these groups, 15 showed a significant difference in abundance between healthy individuals and IBD patients, per Fisher’s exact test (Figure 1C and Supplemental Table 2). Ten groups were more common in healthy individuals, while 5 were predominant in IBD patients. All 5 IBD-enriched clusters were present in both CD and UC patients (Supplemental Table 2). When we analyzed these patient groups separately, 3 of the 5 IBD-enriched clusters reached statistical enrichment in UC (vs. healthy controls) and 2 reached enrichment in CD (Supplemental Figure 2A). However, UC-CD differences were not significant (2-sided Fisher’s per cluster, all *P* ≥ 0.14), indicating comparable enrichment magnitudes across IBD subtypes (Supplemental Table 2B). A CDR3β sequence-similarity t-distributed stochastic neighbor embedding (t-SNE) analysis further supported this shared disease-skewed TCR landscape, showing overlapping UC- and CD-enriched regions relative to healthy controls (Supplemental Figure 3).

We next assessed α chain heterogeneity within β-defined GLIPH clusters using the single-cell subset with paired α-β TCRs. TCRs were embedded by CDR3 similarity using (a) β chain only and (b) paired α-β information (Supplemental Figure 2B). As expected, β chain–defined GLIPH clusters formed tight groups; with α-β information, most clusters remained cohesive while a few partially dispersed, indicating some α chain heterogeneity. We then reclustered with TCRdist (α-β) (Supplemental Figure 2C): 8 of 9 β-defined GLIPH groups achieved purity = 1.0 (all members co-clustered in the dominant TCRdist cluster), and 1 group (E%QET) showed purity = 0.667. Overall agreement between β-defined GLIPH groups and α-β-TCRdist clusters was relatively high (adjusted Rand index = 0.835). Consistent with TCR–peptide-MHC structures, CDR3β nearly always contacts peptide-MHC, whereas CDR3**α** contacts can be absent (16). Moreover, TCRβ exhibits more than 10 times greater sequence diversity than TCR**α** (18), so β chain CDR3 motifs often dominate specificity, while the α chain can provide important refinements (19). Notably, 3 of 5 IBD-associated specificity groups (%IGSGANV, SESG%GQP, and S%LDGYE) contained expanded clones (≥3 identical TCRs; Table 2, Table 3, and Supplemental Table 3), consistent with antigen-driven expansion.

Next, we examined the dominant HLA-DRB1 allele, defined as the most frequently shared allele within each TCR specificity group. Notably, shared HLA alleles were considered at the final prioritization step (Figure 1B), and the “dominant” DRB1 was then assigned post hoc as the most frequent (and allele-wise most significant) within each retained group and is used descriptively below. The DRB1*12:01 risk allele was dominant in 2 IBD-enriched groups (Figure 1D), emphasizing a strong risk allele HLA association with these TCRs. HLA-DRB1 risk alleles dominated all 5 IBD-enriched TCR specificity groups and were absent in the 10 healthy-enriched groups, providing additional evidence for their enrichment in IBD. In contrast, 7 healthy-enriched TCR specificity groups were associated with non-risk alleles (Figure 1D). Supplemental Table 3 further describes each study participant, their HLA-DRB1 allelic composition, and their involvement in the GLIPH-identified clusters. Taken together, these results demonstrate that specific HLA risk alleles are uniquely enriched in IBD.

When we analyzed the sample origins of TCRs from the 5 IBD-associated groups, we found them solely in IBD patients (Figure 1F), spread across both CD and UC patients, regardless of whether the disease was active or in remission (Figure 1, F and G). Interestingly, some patients had TCRs in multiple IBD-prevalent groups, suggesting that these individuals might have broader TCR responses (Figure 1F).

We then wanted to understand whether the 5 IBD-enriched specificity groups we identified in our cohort were present in other IBD cohorts. Indeed, when we performed GLIPH2 analysis on an external, European cohort of 32 IBD patients and 24 healthy controls, we were able to reproduce all 5 IBD-enriched specificity groups (20). Intriguingly, 2 of these, the %IGSGANV motif (OR = 7.25; FDR-adjusted *P* = 0.002) and the RD%LYG motif (OR = 7.25; FDR-adjusted *P* = 0.002), were also significantly enriched in IBD compared with healthy controls (Figure 1E). While the 10 groups common in healthy individuals were also present, they were not enriched in healthy controls to the same degree as in our study. These findings provide further evidence that TCRs in IBD-prevalent groups may recognize a common antigen responsible for the disease, particularly among patients with HLA-DRB1 risk alleles.

Moreover, we next investigated whether these 5 IBD-enriched specificity groups were principally associated with HLA allelic expression, which may be biased toward the alleles more prevalent among IBD patients because of their genetic enrichment in IBD. To address this bias, we spiked in 32,760,689 TCRs from 224 healthy individuals with the IBD risk alleles HLA-DRB1*12:01 (*n* = 20 individuals), HLA-DRB1*11:01 (*n* = 69 individuals), and HLA-DRB1*07:01 (*n* = 151 individuals), sourced from 2 publicly available datasets (21, 22). Re-running GLIPH2 with these TCRs included, we found that 4 of 5 IBD-associated CDR3β motifs were detectable in HLA-DRB1–matched spike-in healthy repertoires. However, in healthy donors, these motif-bearing TCRs showed no statistically significant TRBV (V-gene) enrichment (Fisher’s exact test), unlike in IBD patients (Supplemental Figure 4). Notably, the IBD-associated motif %IGSGANV was also not detected in any healthy individual in these external datasets. Together with shared CDR3β motifs, the significant TRBV enrichment observed in IBD repertoires supports convergent selection and increases the likelihood of shared, HLA-restricted antigenic recognition among these TCRs.

### Identification of cytotoxic CD4^+^ T cells in a subset of IBD patients.

**Figure 2:**
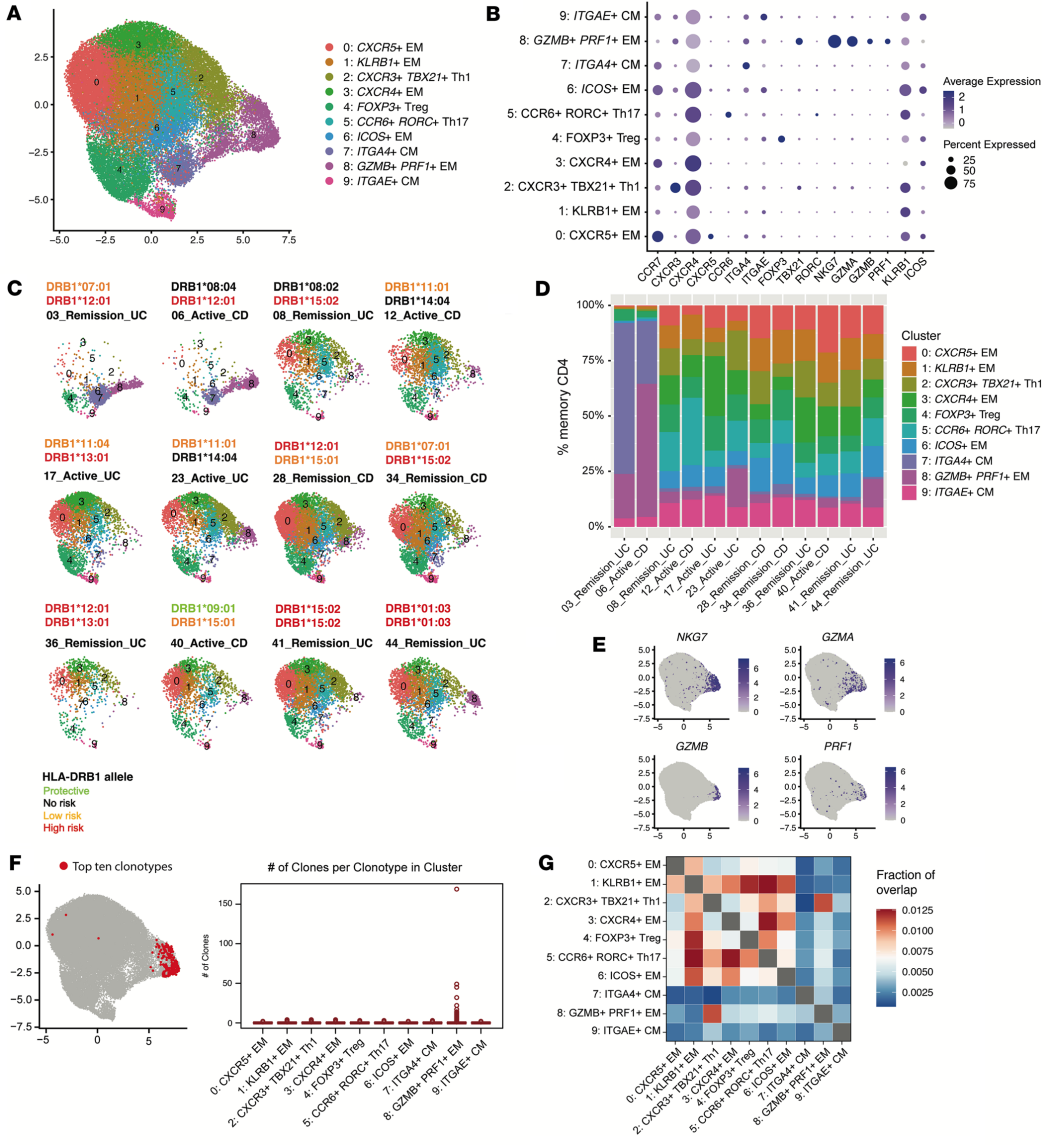
A subset of patients contains clonally expanded, granzyme- and perforin-expressing memory CD4^+^ T cells. (**A**) UMAP of memory CD4^+^ T cell populations identified in a subset of 12 patients. (**B**) Dot plot illustrating gene marker expression of each cell type. (**C**) UMAP of memory CD4^+^ cell types per patient. Individual HLA alleles are colored red if they were risk alleles for both UC and CD, orange if they were a risk allele for either UC or CD, black if they were a risk allele for neither UC nor CD, and green if they were protective for either UC or CD. High risk indicates global IBD, whereas low risk indicates subtype-specific IBD. (**D**) Cell types identified in each patient; each cell type is represented as a percentage of the total memory CD4^+^ T cells sequenced from that patient. (**E**) UMAP expression of the cytotoxic markers *NKG7*, *GZMA*, *GZMB*, and *PRF1*. (**F**) UMAP localization of top 10 expanded clonotypes, colored in red. Also shown is the number of clones per clonotype in each cell population. (**G**) Comparison of TCR repertoire overlap among memory CD4^+^ T cell subsets.

Upon identifying specific TCR groups linked with prevalent HLA-DRB1 risk alleles in certain IBD patients, we next characterized the T cell phenotypes associated with these factors. To achieve this, we used paired single-cell transcriptomics and TCRαβ sequencing analysis on memory CD4^+^ T cells (CD3^+^CD4^+^CD45RA^–^) from the PBMCs of 12 patients, sorted by flow cytometry. These patients were selected because of their HLA-DRB1 risk alleles, expanded T cell populations, and membership in TCR specificity groups associated with IBD (Figure 2).

Cluster analysis revealed 10 distinct memory CD4^+^ T cell clusters. These included 7 *CCR7^–^* effector memory (EM) subsets, 2 *CCR7^+^* central memory (CM) subsets, and one *FOXP3*^+^ regulatory T cell (Treg) subset (Figure 2A). Within the EM subsets, we identified a Th1 population marked by *CXCR3* and *TBX21* (T-bet) and a Th17 population characterized by *CCR6* and *RORC* (RORγT). We noted additional heterogeneity, with varying expression of chemokine receptors (*CXCR4*, *CXCR5*), integrins (*ITGA4*, *ITGAE*), and activation markers (*KLRB1*, *ICOS*, *NKG7*, *GZMA*, *GZMB*, and *PRF1*) across the clusters (Figure 2B).

Compared with the other patients, we observed a marked difference in the distribution of memory CD4^+^ T cell clusters in patients 03 and 06, who both carried the DRB1*12:01 risk allele (Figure 2, C and D). Notably, patients 28 and 36, carrying the same risk allele, displayed a different T cell cluster profile. Four patients (03, 06, 23, and 44) had a notable fraction of memory CD4^+^ T cells in cluster 8, expressing high levels of cytotoxic molecules such as granzymes A and B (*GZMA*, *GZMB*) and perforin (*PRF1*), and thus clearly cytotoxic CD4^+^ T cells (Figure 2, D and E), detailed further in Supplemental Table 4. Within this group, patient 44 uniquely possessed the significant IBD high-risk HLA-DRB1*01:03 allotype (10) in both alleles as well as single-cell paired TCRαβ chains. These cytotoxic CD4^+^ T cells were also significantly clonally expanded, encompassing the majority of the dominant clonotypes in our study (Figure 2F).

We then quantified the extent to which clonotypes were shared across different cell types (Figure 2G). The cytotoxic *GZMB^+^PRF1^+^* subset in cluster 8 primarily shared clonotypes with Th1 cells in cluster 2. Notably, both populations expressed *TBX21* (T-bet), a Th1 lineage–defining transcription factor (Figure 2B). These findings may suggest a differentiation pathway from *CXCR3^+^TBX21^+^* Th1 cells to a cytotoxic *GZMB^+^PRF1^+^* phenotype involved in the pathogenesis of IBD.

### Identification of KIR^+^CD8^+^ T cells in a small subset of IBD with HLA-DRB1 risk.

**Figure 3:**
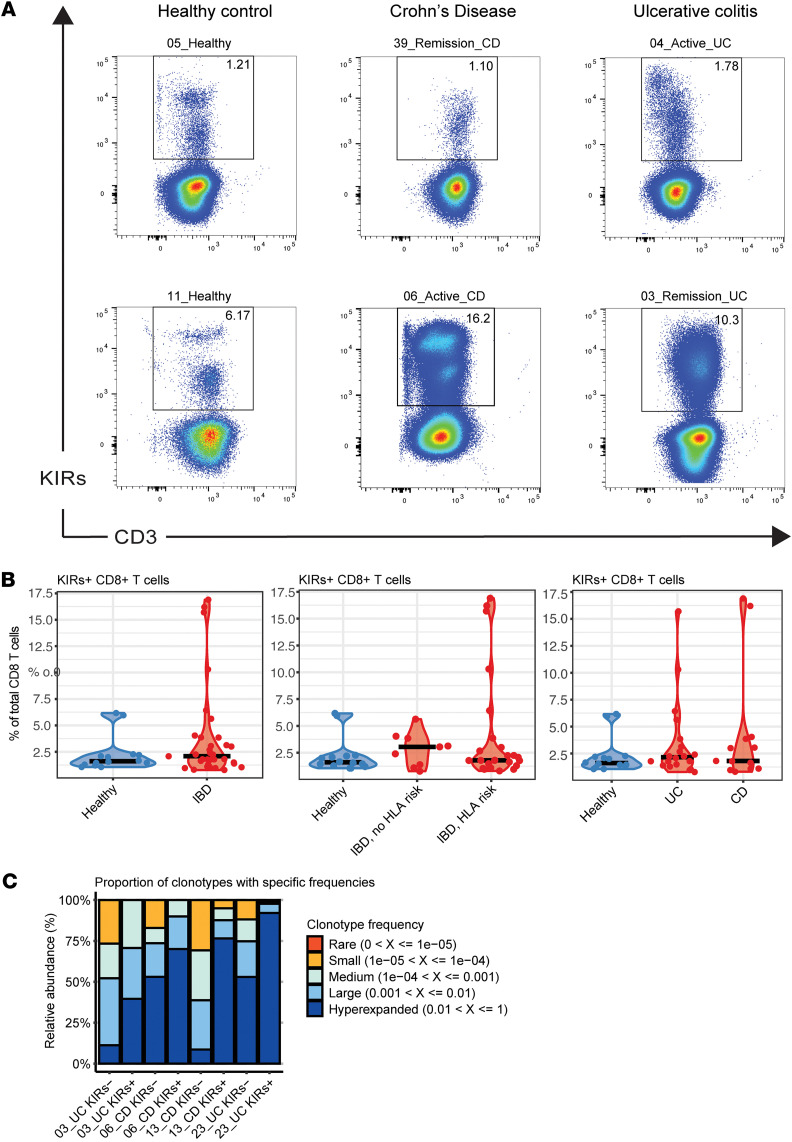
Increased proportion of clonally expanded, KIR^+^CD8^+^ T cells in a subset of IBD patients with HLA-DRB1 risk alleles. (**A**) Representative contour biaxial plots of KIR^+^CD8^+^ T cells in the peripheral blood of healthy controls, CD patients, and UC patients, analyzed by flow cytometry. (**B**) Proportion of KIR^+^CD8^+^ T cells as a fraction of total CD3^+^CD56^–^ T cells, stratified by HLA risk and disease subtype. (**C**) Relative abundance of clonotypes in KIR^–^CD8^+^ versus KIR^+^CD8^+^ T cells of the same patient. Clonotype frequency is defined as the size of clonotypes as a fraction of the total sample repertoire.

Recently we found that a particular subset of CD8^+^ T cells with immunosuppressive functions, identified by their expression of killer cell immunoglobulin-like receptors (KIRs), is elevated in the blood of some patients suffering from classical autoimmune diseases, including celiac disease, multiple sclerosis, and systemic lupus erythematosus (23). However, this subset has not been previously studied in the context of IBD. We found that a small group, representing 12.1% (95% CI 3.4%–28.2%) of IBD patients (4 of 33) and divided equally between those with UC and CD, showed higher levels of these KIR^+^CD8^+^ T cells in their blood (Figure 3, A and B). This increase was not observed in healthy controls. Notably, this rise occurred in both active disease and remission in IBD. While not all patients with HLA-DRB1 risk alleles showed an increase in KIR^+^CD8^+^ T cells, the 4 patients who did have this increase also carried these risk alleles. Three of these 4 patients with elevated KIR^+^CD8^+^ T cells (03, 06, and 23) also had higher levels of clonally expanded, cytotoxic CD4^+^ T cells. In addition, we identified greater clonotype expansion among KIR^+^CD8^+^ T cells compared with their KIR^–^CD8^+^ T counterparts (Figure 3C). Taken together, these findings suggest a potential link between an increased presence of KIR^+^CD8^+^ T cells, clonal expansion of cytotoxic CD4^+^ T cells, and HLA-DRB1 risk alleles in a subset of patients with IBD.

### Cross-disease analysis of KIR^+^CD8^+^ TCR motifs reveals shared patterns in autoimmune and inflammatory conditions.

Given our findings in IBD patients, we examined whether the KIR^+^CD8^+^ TCR repertoire in these IBD patients was similar to those in other autoimmune and inflammatory conditions (23). We thus conducted an integrated GLIPH2 analysis on TCR sequences of KIR^+^CD8^+^ T cells from our IBD patient cohort together with a publicly available independent cohort comprising various autoimmune disease and COVID-19 patients (*n* = 49) and healthy controls (*n* = 17) (Supplemental Figure 5) (23). Three KIR^+^CD8^+^ TCR specificity groups met stringent filtering criteria, including V-segment bias, CDR3 length bias, memory repertoire bias, and shared HLA-I alleles (Supplemental Figure 5). These groups contained exclusively disease-associated TCRs, with 1 group enriched in IBD and 2 spanning multiple diseases.

Notably, the SL%YE specificity group was identified in both a remission UC and a celiac disease patient with identical HLA-A, HLA-B, and HLA-C alleles (Supplemental Table 5). In addition, the S%DSGGTDT specificity group included KIR^+^CD8^+^ TCRs from patients in remission as well as active UC, type 1 diabetes, and COVID-19 patients, with a shared CDR3β sequence, CASSFDSGGTDTQYF, from IBD, type 1 diabetes, and COVID-19 patients (Supplemental Table 5). These findings raise the possibility of shared antigenic responses across diseases.

### Investigating candidate antigens that bind to IBD-associated TCRs.

To investigate the antigen sources that may drive expansion of the IBD-enriched TCRs, we next analyzed 8 public IBD datasets (53,719 TCRs) (20, 24–30) and 3 curated antigen databases (VDJdb, McPAS-TCR, and TBAdb; 68,212 TCRs) (31–33) in which disease traits or antigen reactivities were experimentally defined. We used these resources to see whether our IBD-associated CD4^+^ T cell specificity groups might be related to known viral, commensal, and food-derived antigens. The characteristics of all IBD-associated TCR specificity groups with database-matched antigen specificities are described in Tables 2, 3, 4, and 5. It is important to note that shared TCRβ sequence matches alone do not establish shared antigen specificity, as the TCRα chain often makes a critical contribution to antigen recognition (19). Nevertheless, this analysis identifies leads (e.g., influenza A, *Candida*, and *Saccharomyces*) that may be important in identifying the causal antigens in IBD. Complementing these in silico links, we quantified circulating *Candida albicans*–reactive CD4^+^ T cells using a CD154 upregulation assay (13) in 5 independent donors (Supplemental Figure 6). Stimulation with heat-killed *C*. *albicans* induced CD154^+^CD4^+^ T cells, and this response was markedly reduced by HLA-DR blockade (and by a pan–DR/DP/DQ antibody), with minimal effect of HLA-DQ or -DP blockade, consistent with predominant HLA-DR restriction.

Taken together, our data indicate that a subset of IBD-enriched TCR β chain specificity groups may relate to known viral and yeast antigen specificities, revealing a degree of reactivity that might sustain dysregulated T cell responses in IBD. One plausible interpretation is that CD4^+^ T cells contribute by recognizing overlapping antigens across these sources. Nonetheless, the precise antigens and cellular reservoirs remain to be further defined, and causal testing in larger, HLA-matched cohorts is warranted to pinpoint drivers of inflammation.

## Discussion

In this study, we related HLA-DRB1 genotype to memory CD4^+^ T cell repertoires in IBD. IBD patients carried a higher burden of HLA-DRB1 risk alleles, and GLIPH2 clustering revealed IBD-enriched TCR specificity groups with clonal expansion and associations with HLA class II risk alleles. The fact that both CD and UC patients shared all 5 of these specificity groups indicates that they target the same small group of antigens. Leveraging public IBD repertoires and curated antigen databases, we mapped 4 of the 5 groups to plausible viral/yeast antigens, providing functional context for their enrichment. A subset of risk-allele carriers also showed expanded cytotoxic *GZMB^+^PRF1^+^*CD4^+^ T cells and KIR^+^CD8^+^ T cells in peripheral blood. These common antigen-specific T cell responses are likely contributing to HLA-driven IBD pathogenesis.

Moreover, the traditional classification of IBD into CD and UC does not fully capture its complexity. Increasing evidence shows substantial overlap in clinical features, genetic risk factors, immunological profiles, and microbial characteristics between CD and UC (15, 34–37), suggesting that these diseases may represent points on a spectrum rather than distinct entities. Our findings also corroborate previous findings that specific HLA-DRB1 alleles are key genetic factors associated with both CD and UC. These alleles have been shown to both influence the risk of developing IBD and contribute to its clinical course and severity (11, 38–41). For example, HLA-DRB1 subtypes have been linked to early-onset, aggressive disease progression and complications such as strictures and fistulas in CD (42). This underscores the critical role of HLA-DRB1 variants in IBD pathogenesis and highlights the potential of tailoring diagnosis and therapy to individual immunogenetic profiles.

Previous studies have highlighted a notable presence of activated HLA-DR^+^CD38^+^CD4^+^ T cells in the intestinal tissue of IBD patients, mainly where inflammation is concentrated near HLA-DR–expressing intestinal epithelium (15). The expression of HLA-DR by intestinal epithelial cells has been correlated with the severity of disease in IBD (43). Future research should investigate the T cell repertoire in relation to HLA-DRB1 risk variants within the intestinal context.

Prior studies have also reported TCR repertoire differences between IBD patients and healthy controls. For instance, a recent twin study revealed that IBD patients exhibited reduced TCRβ chain diversity compared with their healthy twins (20). Another study reported decreased intestinal TCR repertoire diversity in CD patients compared with healthy controls. While our research focused on blood samples, it underscores the importance of evaluating TCR diversity, abundance, and sharing in the intestinal tissue of IBD patients relative to healthy individuals.

The vast diversity of the TCR sequence repertoire poses a major challenge to identifying shared antigenic specificities across patients. Here, we used the GLIPH2 algorithm (16, 17), which grouped over 3 million TCR sequences by conserved patterns in their CDR3 amino acid sequences, crucial for antigen recognition. This computational approach has previously proven effective in identifying disease-relevant T cells in autoimmune disorders (44), infectious diseases (17), and tumor immunology (45). GLIPH2 allowed us to identify 5 TCR specificity groups unique to IBD, with 3 showing clonal expansion, pointing to antigen-driven selection. Because our discovery data lack paired α-β chains for these groups, in vitro TCR cloning for functional testing was not feasible; future α-β capture will enable definitive antigen testing. Notably, all 5 groups were associated with HLA-DRB1 risk alleles (07:01, 11:01, and 12:01), suggesting a potential uniform T cell response to shared antigens presented by these class II molecules, consistent with shared groove properties (9).

Cytotoxic EM CD4^+^ T cells (GZMB^+^PRF1^+^) were clonally expanded in a subset of risk-allele carriers and shared clonotypes with Th1 cells, indicating a potential differentiation path from a Th1 to a cytotoxic phenotype in IBD. This aligns with previous findings of intestine-derived Th1 cell clones in CD patients expressing the cytotoxic protein GZMB in vitro (46). Earlier studies have also identified increased cytotoxic CD4^+^ T cells in the intestines of UC and CD patients (46–52). In CD, these cytotoxic CD4^+^ T cells can react to commensal yeast, suggesting their involvement in immune responses to gut microbiota (30). Furthermore, mouse models of acute graft-versus-host disease have found elevated cytotoxic CD4^+^ T cells in inflamed intestinal tissue (53, 54). Our research extends these observations by detecting these cytotoxic CD4^+^ T cells in the peripheral blood of patients with HLA-DRB1 risk alleles, which may offer new insights into systemic immune responses in IBD.

We also observed co-occurrence of elevated KIR^+^CD8^+^ T cells with cytotoxic CD4^+^ T cells in a subset of patients (4/33 overall; 3/4 overlapping), echoing patterns across multiple autoimmune diseases: KIR^+^CD8^+^ T cells in celiac disease, lupus, and multiple sclerosis, where they were also elevated and capable of modulating inflammatory immune responses (23); and cytotoxic CD4^+^ T cells in celiac disease, multiple sclerosis, and rheumatoid arthritis (55–57), supporting an autoimmune-like endotype that may explain part of IBD heterogeneity. Consistent with cross-disease convergence, our integrated analysis of KIR^+^CD8^+^ T cell repertoires identified shared TCR sequence motifs across IBD and other conditions. One group (S%DSGGTDT) contained an identical CDR3β sequence in IBD, type 1 diabetes, and COVID-19, and another (SL%YE) was shared between a remission UC patient and a celiac disease patient with identical HLA class I alleles. Whether these populations are pathogenic or protective remains unresolved, particularly given data in celiac disease linking higher suppressive KIR^+^CD8^+^ levels to active disease (23). By analogy, while KIR^+^CD8^+^ cells HLA-I–dependently kill gliadin-specific CD4^+^ T cells, we hypothesize that KIR^+^CD8^+^ cells in IBD recognize class I–presented self-peptides on activated CD4^+^ T cells, potentially clonotypic TCR-derived or activation/stress-induced peptides, providing a mechanistic basis for our cross-disease KIR^+^CD8^+^ GLIPH motifs. Our CD45RA^–^ gating limited KIR^+^CD8^+^ analysis to EM/CM cells; future work should resolve CD45RA^+^ virtual memory subsets and test function.

Across UC and CD, the 5 TCR specificity groups were similarly prevalent, arguing against subtype-restricted antigens. All 5 were enriched among HLA-DRB1 risk alleles, and, after we spiked in 32.76 million TCRs from 224 HLA-matched healthy donors, 4 motifs were detectable without TRBV enrichment, whereas %IGSGANV was absent in all healthy donors. In IBD, by contrast, these motifs showed significant TRBV enrichment, supporting convergent selection. Two of 5 groups were also enriched in an independent IBD cohort (20).

To connect specificity with antigenic drivers, we screened 8 IBD repertoires (20, 24–30) and curated antigen databases (VDJdb, McPAS-TCR, and TBAdb) (31–33). This prioritized candidates for 4 of 5 IBD-enriched groups: %IGSGANV to influenza *M1* and *Candida* *tropicalis* (consistent with potential cross-reactivity), RD%LYG and %RGATGE to *C*. *albicans*, and S%LDGYE to *Saccharomyces* *cerevisiae*, while SESG%GQP remains unmatched. We emphasize that these matches are β chain–based and hypothesis-generating, not proof of specificity; public databases are enriched for viral epitopes and undersample many gut antigens. Nonetheless, the candidate assignments align with literature: the gut mycobiome shows *Candida* increases in both CD and UC (58), with stronger antifungal immunity in CD (e.g., higher anti–*S*. *cerevisiae* antibodies) (59–61), yet yeast-reactive CD4^+^ T cells are reported in both IBD subtypes (30, 62), mirroring our cross-disease motifs. Moreover, we detected circulating HLA-DR–restricted *C*. *albicans*–reactive CD154^+^CD4^+^ T cells by HLA blockade assays, in line with reports that HLA-DRB1–matched fungus-specific T cells mediate antifungal activity (63). Conversely, influenza imprinting provides a ubiquitous viral memory reservoir: the M1 protein carries conserved, DR-restricted CD4^+^ epitopes, and influenza can perturb the gut (64–66); given frequent T cell cross-reactivity (67,68), influenza-primed CD4^+^ cells may recognize microbial peptides in the intestine. Consistent with this, our %IGSGANV motif includes TCRs matching both influenza A *M1* and *C*. *tropicalis*, suggesting one route to shared responses. This is likely the tip of the antigenic iceberg: bacterial epitopes (e.g., Lachnospiraceae flagellins) also elicit CD4^+^ responses and become inflammatory in IBD (13, 69). While these data outline plausible pathways linking microbiome and viral exposures to IBD pathology, definitive antigen assignment will require paired α-β identification, TCR reconstitution, and HLA-matched testing.

Together, these convergent features (cross-disease presence, HLA-II risk bias, clonal expansion, absence or lack of V-gene enrichment in HLA-matched healthy controls, and antigen-linked matches) support these 5 β-motif clusters as biologically meaningful in IBD and validate our TCR findings across larger, independent datasets. The classification of IBD as an autoimmune disease remains controversial (70–73). However, our data strongly suggest that HLA class II–driven IBD exhibits autoimmune-like features in a subset of patients, supported by (a) associations with specific HLA class II alleles, (b) the identification of TCR specificity groups linked to these risk alleles, (c) the presence of cytotoxic CD4^+^ T cells, and (d) elevated regulatory KIR^+^CD8^+^ T cells, as well as shared TCRs from those T cells, previously implicated in multiple classical autoimmune disorders (23). Taken together, these findings support the view that IBD exhibits autoimmune-like features in a subset of patients.

## Methods

### Sex as a biological variable.

Peripheral blood samples for flow cytometry, RNA sequencing, TCR sequencing, and HLA typing were obtained from 23 males (48.9%) and 24 females (51.1%).

### Human samples.

Frozen PBMC samples from 14 healthy controls, 20 UC patients, and 13 CD patients were obtained from Stanford Hospital. The study was approved by the Stanford University Administrative Panels on Human Subjects in Medical Research and covered by IRB 61585. All participants provided informed written consent.

### HLA typing and risk classification.

We performed next-generation-based HLA genotyping with the AlloSeq Tx17 system (CareDx) using genomic DNA extracted from PBMCs of study participants. HLA alleles were analyzed using AlloSeq Assign Software and classified as risk alleles if they had an odds ratio greater than 1, deduced from prior studies, for either CD or UC (9).

### Flow cytometric analysis.

Frozen PBMC samples were thawed in 50% FBS, 50% RPMI culture medium and washed with FACS buffer (phosphate-buffered saline [PBS] plus 0.1% bovine serum albumin [BSA], 0.05% sodium azide, and 2 mM EDTA). Then, samples were centrifuged at 475*g* and treated with Fc receptor block (BioLegend; 10 μg/mL) in FACS buffer (0.5% BSA, 2 mM EDTA in PBS) followed by staining with antibodies against surface molecules (30 minutes, 4°C). The following antibodies were obtained from BioLegend: CD3 (clone UCHT1; catalog 300412; 1:100), CD4 (clone RPA-T4; catalog 300532; 1:100), CD8a (clone RPA-T8; catalog 301042; 1:100), CCR7 (clone G043H7; catalog 353212; 1:10), KIR2DL2/L3/S2 (clone Dx27; catalog 312602; 1:100), KIR3DL1 (clone Dx9; catalog 312706; 1:100), and KIR2DL5 (clone UP-R1; catalog 347002; 1:100). The antibody CD45RA (clone HI100; catalog 562298; 1:300) was sourced from BD Biosciences. KIR2DL5 (catalog 143211; 1:100) was obtained from R&D Systems. The cells were then analyzed with an LSR II instrument or sorted on a FACSAria II instrument (BD Biosciences) and analyzed with FlowJo version 10.10.0 (BD Biosciences). Dead cells were excluded based on viability dye staining (LIVE/DEAD Fixable Blue Dead Cell Stain Kit, Invitrogen, catalog L34962). FlowJo software was used for visualization of cell frequencies, and comparisons were performed using the R package ggplot2.

### Bulk TCRβ sequencing.

Memory CD4^+^ T cells were flow cytometry–sorted from PBMCs of 14 healthy controls, 13 CD patients, and 20 UC patients, followed by extraction of genomic DNA using the QIAGEN QIAamp DNA Blood Mini Kit. Next, the immunoSEQ assay (Adaptive Biotechnologies) was performed to sequence the CDR3 regions of TCRβ chains from genomic DNA (74). Subsequently, the R package immunarch was used to compare the relative abundance and diversity of clonotypes in the bulk TCR repertoire data of healthy controls and IBD patients (75).

### GLIPH2 filtering.

In brief, GLIPH2 computes several significance metrics for each candidate specificity group: (a) a V-segment bias (Vb) score assessing whether a cluster’s TCRs over-utilize a particular V gene, (b) a CDR3 length score assessing whether the cluster could arise from TCRs simply sharing similar lengths, (c) a Fisher’s exact score evaluating enrichment of the cluster in one repertoire versus a reference (in our case, memory vs. naive T cell repertoires), and (d) an HLA association score testing whether the cluster’s sequences are enriched among individuals sharing an HLA allele. We used GLIPH2’s default significance cutoffs (Vb score *P* < 0.05, CDR3 length *P* < 0.05, Fisher’s enrichment *P* < 0.1, and HLA association *P* < 0.05) to obtain a high-confidence set of 440 specificity groups. A Vb score less than 0.05, for instance, refers to a *P* value indicating non-random clustering by V-gene usage; by requiring this to be below 0.05, we excluded clusters that could be artifactual due to shared V-gene preference rather than true CDR3 motif similarity.

### Single-cell TCR and RNA sequencing.

Paired single-cell TCR and RNA sequencing was performed on flow cytometry–sorted memory CD4^+^ T cells from PBMCs of a subset of patients, specifically 5 with CD and 7 with UC, using BD Rhapsody technology (BD Biosciences), in accordance with the manufacturer’s instructions. In brief, cryovials were thawed in medium (50% FCS/50% RPMI) at 37°C and stained with the antibodies CD3 (clone UCHT1; BioLegend), CD4 (clone RPA-T4; BioLegend), CD8a (clone RPA-T8; BioLegend), and CD45RA (clone HI100; BD Biosciences) and viability dye (Invitrogen). Approximately 30,000 cells were sorted per sample. Subsequently, samples were stained with oligonucleotide-conjugated Sample Tags from the BD Human Single-Cell Multiplexing Kit in BD stain buffer following the manufacturer’s protocol. Barcoded samples were then washed and spun down at 350*g* for 10 minutes and pooled. The pooled sample was then stained concurrently with oligonucleotide-conjugated antibody cocktail (BD Biosciences; custom TotalSeq-C oligonucleotide-conjugated panel; custom table). Staining was in BD stain buffer for 30 minutes on ice, and samples were then spun down at 350*g* for 10 minutes and washed 3 times. Pellet was resuspended in Rhapsody buffer for capture. Cell capture and library preparation were completed using the BD Rhapsody Targeted mRNA Kit. Cells were captured with beads in a microwell plate, followed by cell lysis, bead retrieval, cDNA synthesis, template switching, Klenow extension, and library preparation in the Stanford Human Immune Monitoring Center following the BD Rhapsody protocol. Libraries were created for TCRs, sample tags, and targeted mRNA using the human T cell panel. Sequencing was completed on NovaSeq (Illumina) in the Human Immune Monitoring Center. Rhapsody data were processed using the Seven Bridges Genomics (SBG; now Velsera) online platform and BD Rhapsody Targeted Analysis Pipeline with V(D)J processing incorporated. Individual samples were de-barcoded using SeqGeq (BD Biosciences) software, and cell populations, along with their gene expression profiles, were extracted for further analysis.

The R toolkit Seurat was used to perform quality control and analysis of single-cell transcriptomic Rhapsody data (76). In brief, we embedded the cells through a *k*-nearest neighbor graph and reduced the data with principal component analysis. The Louvain algorithm, a method of modularity optimization, then clustered these cells. We determined the number of cell specificity groups by adjusting the resolution parameter. Then, dimensionality reduction using UMAP was performed on the gene expression data, using the umap package in R.

### GLIPH2 clustering of CDR3β sequences.

Bulk TCRβ sequences of sorted CD45RA^–^CD3^+^CD4^+^ memory T cells (1,936,565 unique TCRs) from healthy controls (*n* = 14), CD patients (*n* = 13), and UC patients (*n* = 20) along with their class II HLA allele compositions were used as data input. GLIPH2 clustering yielded 468,441 specificity groups that were derived from 3 or more individuals and contained 3 or more unique CDR3β sequences; of these, 440 groups were selected based on the following default criteria: Vb score < 0.05, CDR3 length score < 0.05, Fisher’s score < 0.1, and HLA score < 0.05.

### CD154 upregulation microbiota-reactive T cell assay.

Heat-killed lyophilized cells from *Candida albicans* (InvivoGen) were resuspended in sterile, endotoxin-free water, with a final concentration of 1 × 10^8^ cells/mL used in the CD154 microbiota-reactive T cell assay (13). PBMCs from healthy blood bank donors were resuspended in MACS buffer (HBSS/0.5% HS/2 mM EDTA) and labeled with CD14 microbeads (Miltenyi Biotec) to magnetically separate PBMCs into monocyte and lymphocyte fractions according to the manufacturer’s protocol. Monocytes were then seeded at 0.5 × 10^6^ cells per well in a 96-well plate, while lymphocytes were seeded separately at 1 × 10^6^ cells per well, both in culture medium (IMDM/10% HS/1% PS). Before antigen loading, anti–HLA-DR (clone L243; BioLegend; catalog 307602; 20 μg/mL), anti–HLA-DQ (clone Tu169; BioLegend; catalog 318102; 20 μg/mL), anti–HLA-DP (clone B7/21; BioLegend; catalog 362302; 20 μg/mL), or a pan-block (anti–HLA-DR/DQ/DP) was added to the monocyte fraction. Monocytes were incubated with blocking antibodies for 30 minutes at 37°C, after which they were preloaded with *C*. *albicans*. Both fractions were then incubated overnight at 37°C. The following day, the lymphocyte fraction was also incubated with blocking antibodies for 30 minutes at 37°C before lymphocytes and monocytes were combined in coculture. Anti-CD40 (clone HB14; Miltenyi Biotec; catalog 130-094-133; 1 μg/mL) was added at the start of coculture. Cells were incubated overnight at 37°C before harvesting and measured by spectral flow cytometry. This experiment included PBMCs from 5 independent healthy donors across 3 independent experiments.

### Statistics.

We used a range of statistical analyses to evaluate the relationship between HLA-DRB1 alleles, TCR repertoire features, and disease status in IBD. We used χ^2^ tests to assess associations between HLA-DRB1 risk allele categories and disease subtypes (UC, CD, and healthy controls). For TCR repertoire analyses, we used the GLIPH2 algorithm to cluster 3.1 million TCRβ sequences into 468,441 specificity groups, applying filtering criteria including V-segment bias, CDR3 length, and memory repertoire enrichment. Fisher’s exact test identified 15 specificity groups significantly enriched in either IBD patients or healthy controls. Single-cell RNA and TCRαβ sequencing enabled unsupervised clustering (UMAP) of CD4^+^ memory T cells, revealing cytotoxic subsets in high-risk patients, with clonotype expansion analyzed through overlap matrices and visualization of dominant clonotypes. Flow cytometry quantified KIR^+^CD8^+^ T cells across groups, and clonotype frequencies were compared between KIR^+^ and KIR^–^ CD8^+^ subsets. We compared distributions of age, sex, disease duration, location/extent, activity, and treatment between patients with and without cytotoxic CD4^+^ or elevated KIR^+^CD8^+^ expansions. Cross-cohort GLIPH2 analysis was conducted to identify conserved TCR specificity groups in autoimmune and inflammatory disease datasets, with shared motifs assessed in relation to HLA-I alleles and disease phenotype. We also integrated the 8 public IBD TCR datasets (20, 24–30) and the 3 curated antigen databases (VDJdb, McPAS-TCR, and TBAdb) (31–33) with our study dataset using GLIPH2 analysis. Statistical analysis for the *Candida*-reactive T cell assay was performed using a Friedman test, with false discovery rate (FDR) correction applied for multiple comparisons. *P* values less than 0.05 were considered significant.

### Study approval.

The studies involving human participants were reviewed and approved by the Stanford University Administrative Panels on Human Subjects in Medical Research (protocol IRB 61585). All samples were obtained after informed consent and medical ethical commission approval, in accordance with the local ethical guidelines of Stanford University and in accordance with the Declaration of Helsinki. Written informed consent to participate in this study was provided by the participants’ legal guardian or next of kin prior to participation.

### Data availability.

Next-generation sequencing data were deposited in the NCBI’s Gene Expression Omnibus (GEO), a MINSEQE-compliant public repository, under accession numbers GSE330122 (scRNA-seq) and GSE330123 (bulk TCR-seq). Supporting data values associated with the main article and supplemental material can be found in the Supporting Data Values file.

## Author contributions

JEC, VVU, and MMD conceived the study and wrote the manuscript. JEC and VVU performed the experiments with the help of AM, QM, JK, and CL. Also, JEC performed most data analyses with the help of VVU. Moreover, XJ provided conceptual input. BC and SS provided clinical material. All authors discussed the results and commented on the manuscript. All authors contributed to the article and approved the submitted version.

## Conflict of interest

MMD is a scientific cofounder of, member of the scientific advisory board of, paid consultant for, and equity holder in Mozart Therapeutics Inc. This company did not pay for, contribute, have any special access to data from, or have any involvement in any aspect of the work.

## Funding support

This work is the result of NIH funding, in whole or in part, and is subject to the NIH Public Access Policy. Through acceptance of this federal funding, the NIH has been given a right to make the work publicly available in PubMed Central.

- National Institute of Allergy and Infectious Diseases (2U19AI057229).
- The Netherlands Organization for Scientific Research (ZonMW Rubicon 452181214 to VVU).
- HORIZON Marie Skłodowska-Curie Actions Postdoctoral Fellowship (101109788 to VVU).
- Propel a Cure for Crohn’s Disease Research.

## Supplementary Material

Supplemental data

Supporting data values

## Figures and Tables

**Table 1:**
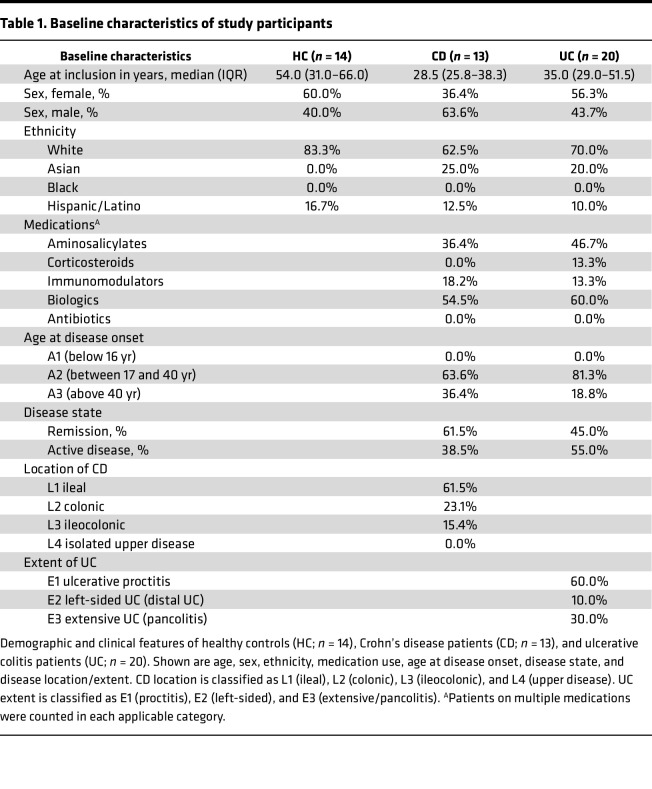
Baseline characteristics of study participants

**Table 2:**
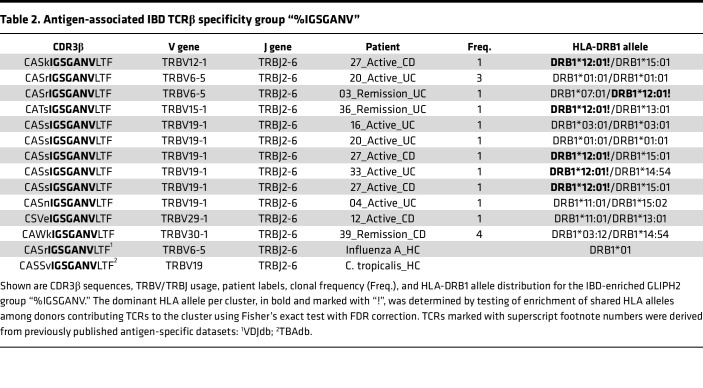
Antigen-associated IBD TCRβ specificity group “%IGSGANV”

**Table 3:**
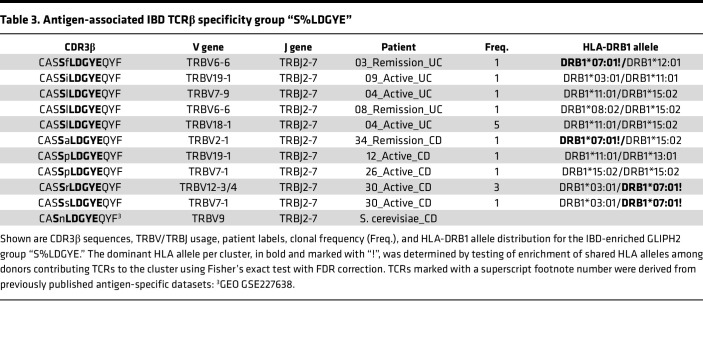
Antigen-associated IBD TCRβ specificity group “S%LDGYE”

**Table 4:**
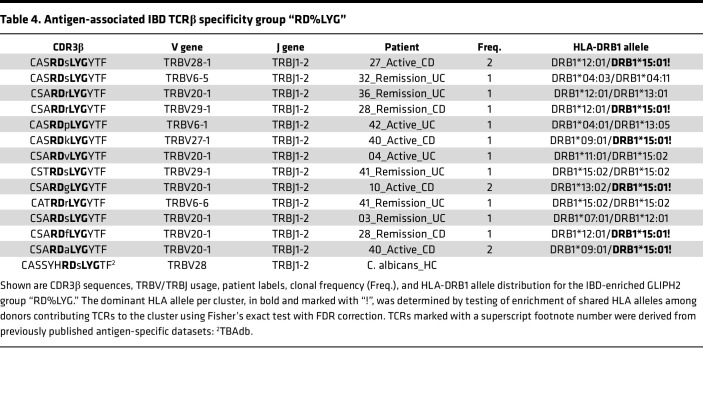
Antigen-associated IBD TCRβ specificity group “RD%LYG”

**Table 5:**
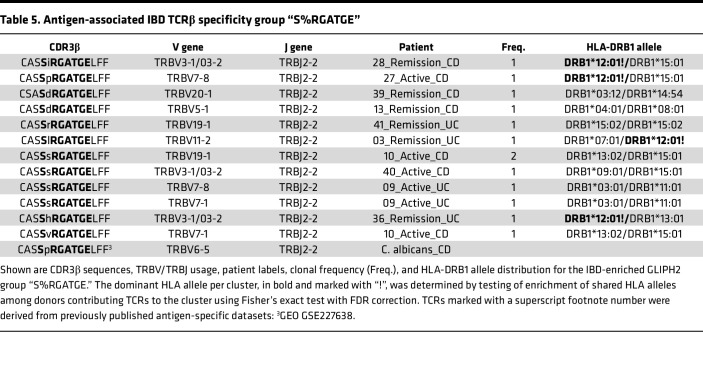
Antigen-associated IBD TCRβ specificity group “S%RGATGE”

## Version 1 06/09/2026

In-Press Preview

## Version 2 08/10/2026

Electronic publication
