# Supplementary material for: Shared CD4+ T cell receptor specificity groups in Crohn’s disease and ulcerative colitis
Source: JCI Insight. 2026 Jun 9;11(15):e195354. doi: 10.1172/jci.insight.195354 (PMC13463618; doi:10.1172/jci.insight.195354)
Supplement: Supplementary file 1 [file jciinsight-11-195354-s212.pdf]

Supplementary Figure 1

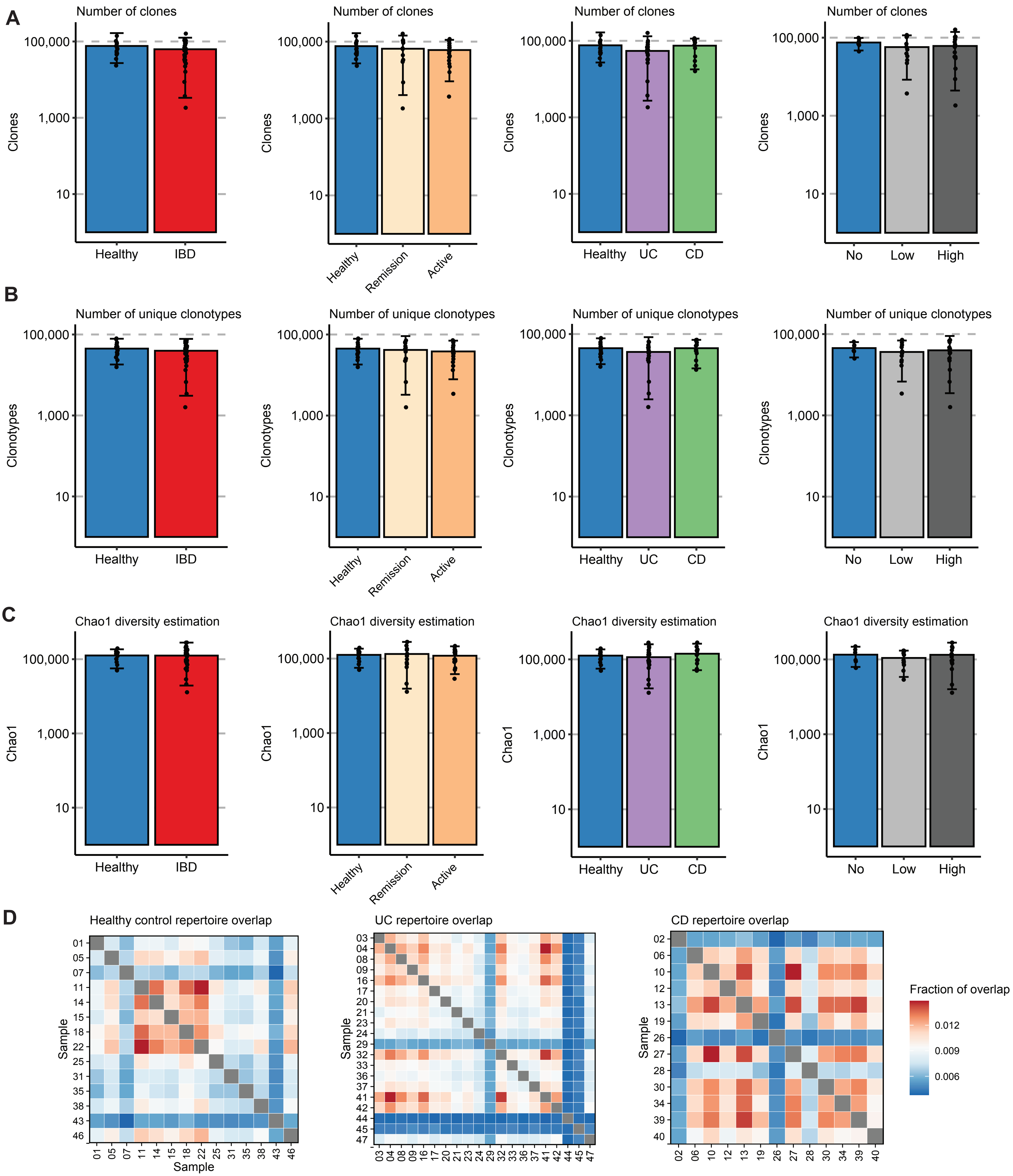

Supplementary Figure 1. CD4+ memory T cell repertoire abundance and diversity are preserved in IBD.
(A–C) Bar graphs showing clone count (A), unique clonotype count (B), and Chao1 diversity index (C) of memory CD4+ T cells, compared across healthy vs. IBD, disease activity (remission vs. active), IBD subtype (UC vs. CD), and HLA-DRB1 risk allele burden (none/low/high). Bars indicate mean; error bars indicate 2.5th–97.5th percentile confidence intervals; dots represent individual samples. (D) Pairwise clonotype overlap heatmaps for healthy control, UC, and CD repertoires. Color scale indicates fraction of overlap.

Supplementary Figure 2

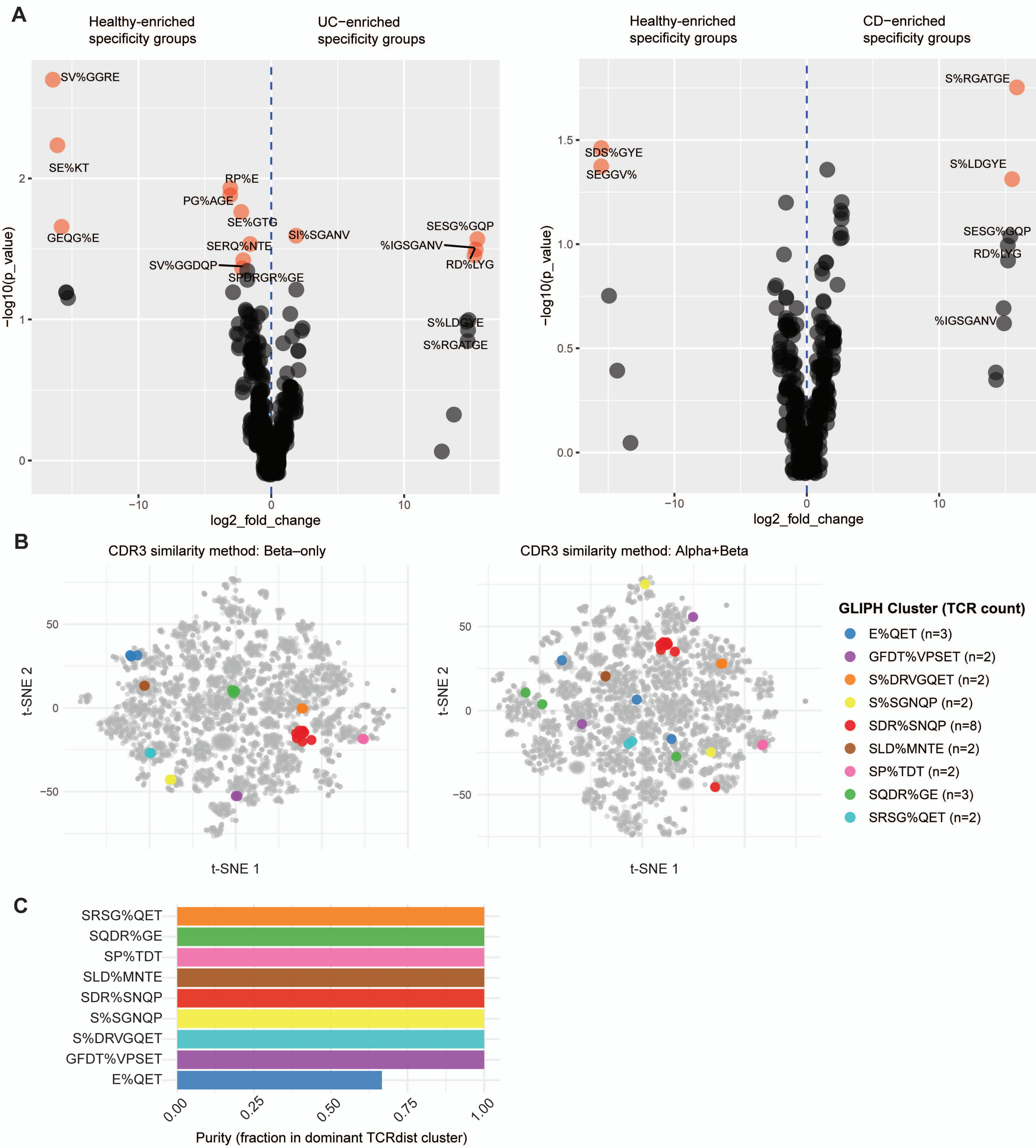

Supplementary Figure 2. CDR3 specificity group analysis using β-chain and α+β-chain clustering.
(A) Volcano plots of GLIPH2 specificity groups enriched in UC (left) or CD (right) versus healthy controls. Log2 fold-change reflects IBD vs. healthy enrichment; significantly enriched groups are highlighted in red. (B) t-SNE visualization of 6,962 paired TCRs based on CDR3β-only (left) or combined CDR3α+β (right) sequence similarity. Colored points denote the largest GLIPH2 clusters (motif label; n = TCR count); gray points are unclustered. (C) Purity (fraction of TCRs within the dominant TCRdist cluster) for each major GLIPH2 specificity group.

Supplementary Figure 3

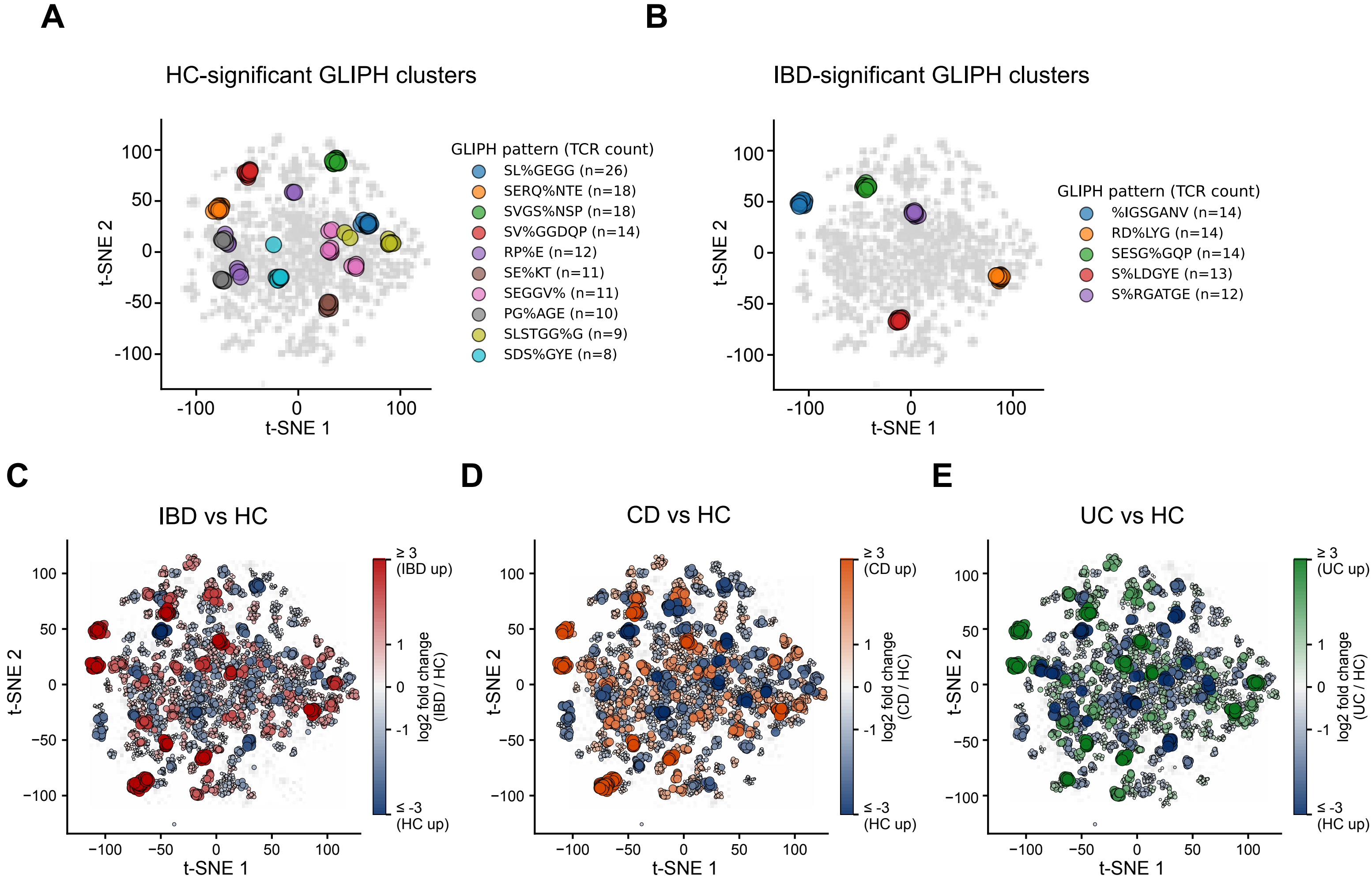

Supplementary Figure 3: CDR3β sequence-similarity t-SNE reveals disease-skewed TCR territories. t-SNE embedding of all 6,915 CDR3β sequences from the GLIPH-clustered IBD cohort, computed on a length-normalised Levenshtein distance matrix (perplexity 20). Each dot represents one TCR; t-SNE 1 / t-SNE 2 axes are identical across panels. (**A**) TCRs belonging to the ten HC-significant GLIPH patterns highlighted on the grey background of all TCRs. Each colour corresponds to one GLIPH pattern; legend gives the pattern motif and the number of TCRs (n). (B) Same map, highlighting TCRs in the five IBD-significant GLIPH patterns. (**C–E**) Per-cluster $\log_2$ fold change of disease vs healthy-control representation, then mapped onto every TCR in that cluster. Diverging colour scales are clipped at ±3 and centred on 0 (no enrichment). (C) IBD vs HC. (D) CD vs HC (E) UC vs HC.

Supplementary Figure 4

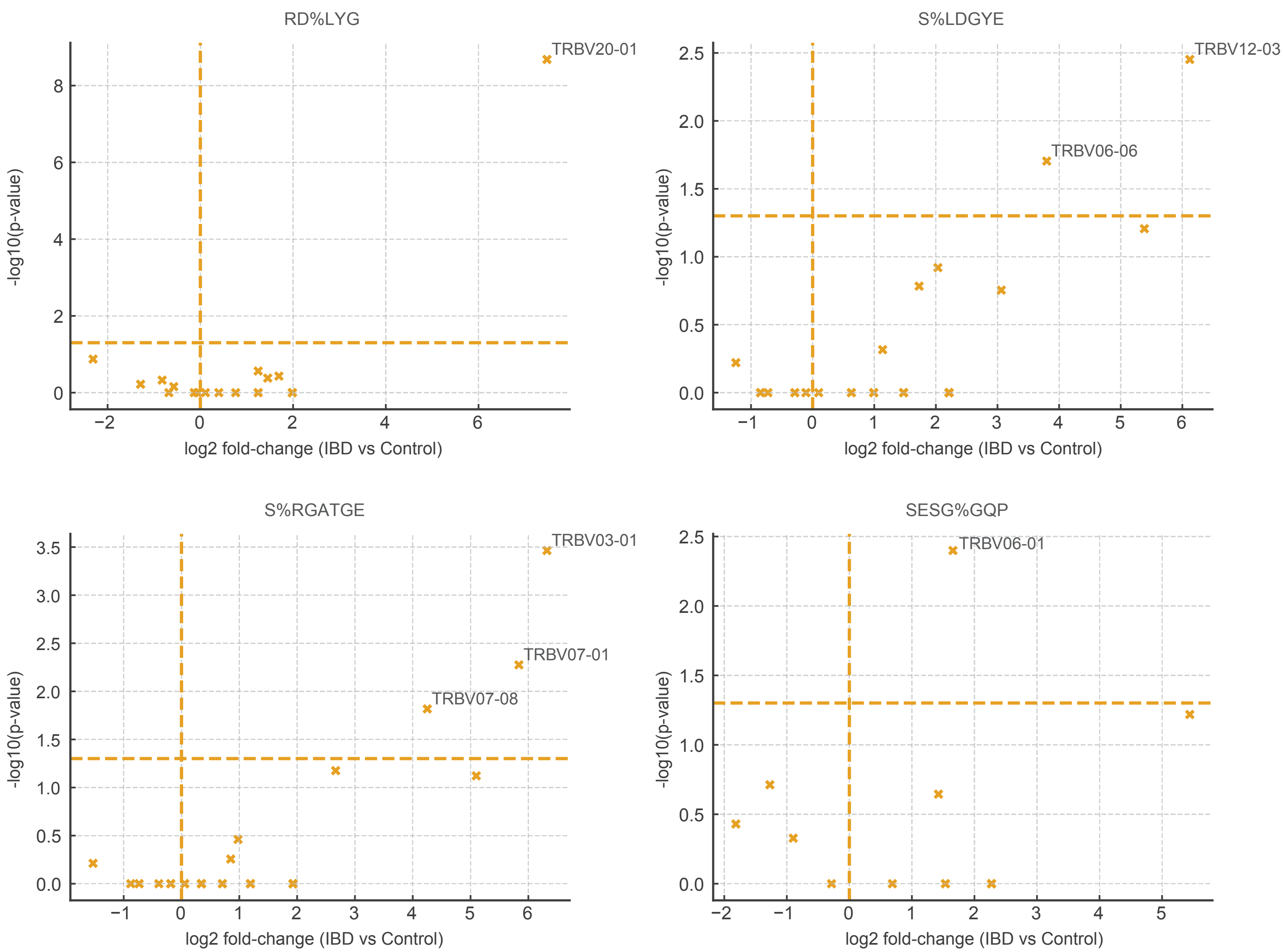

Supplementary Figure 4: Volcano plots showing differential T cell receptor Vβ gene segment usage in IBD versus healthy controls across four CDR3 motif clusters. Each panel represents a distinct CDR3 amino acid motif cluster (RD%LYG, S%LDGYE, S%RGATGE, SESG%GQP). The x-axis displays the $\log_2$ fold-change in TRBV gene segment frequency between IBD patients and healthy controls (positive values indicate enrichment in IBD). The y-axis displays the $-\log_{10}$(p-value) of the association. Each point (×) represents an individual TRBV gene segment. The horizontal dashed line denotes the significance threshold ($p = 0.05$); the vertical dashed line marks $\log_2$ fold-change = 0. TRBV segments exceeding the significance threshold are labeled. Across all four motif clusters, significantly enriched segments are exclusively right-shifted, indicating consistent expansion in IBD relative to controls, with TRBV20-01, TRBV12-03, TRBV03-01, and TRBV06-01 representing the top hits in their respective clusters.

Supplementary Figure 5

Patient	# CDR3β sequences
IBD-03	545
IBD-06	250
IBD-13	1,332
IBD-23	175
Li et al., healthy controls (n=17)	6,268
Li et al., patients with autoimmune disease and COVID-19 (n=49)	6,978

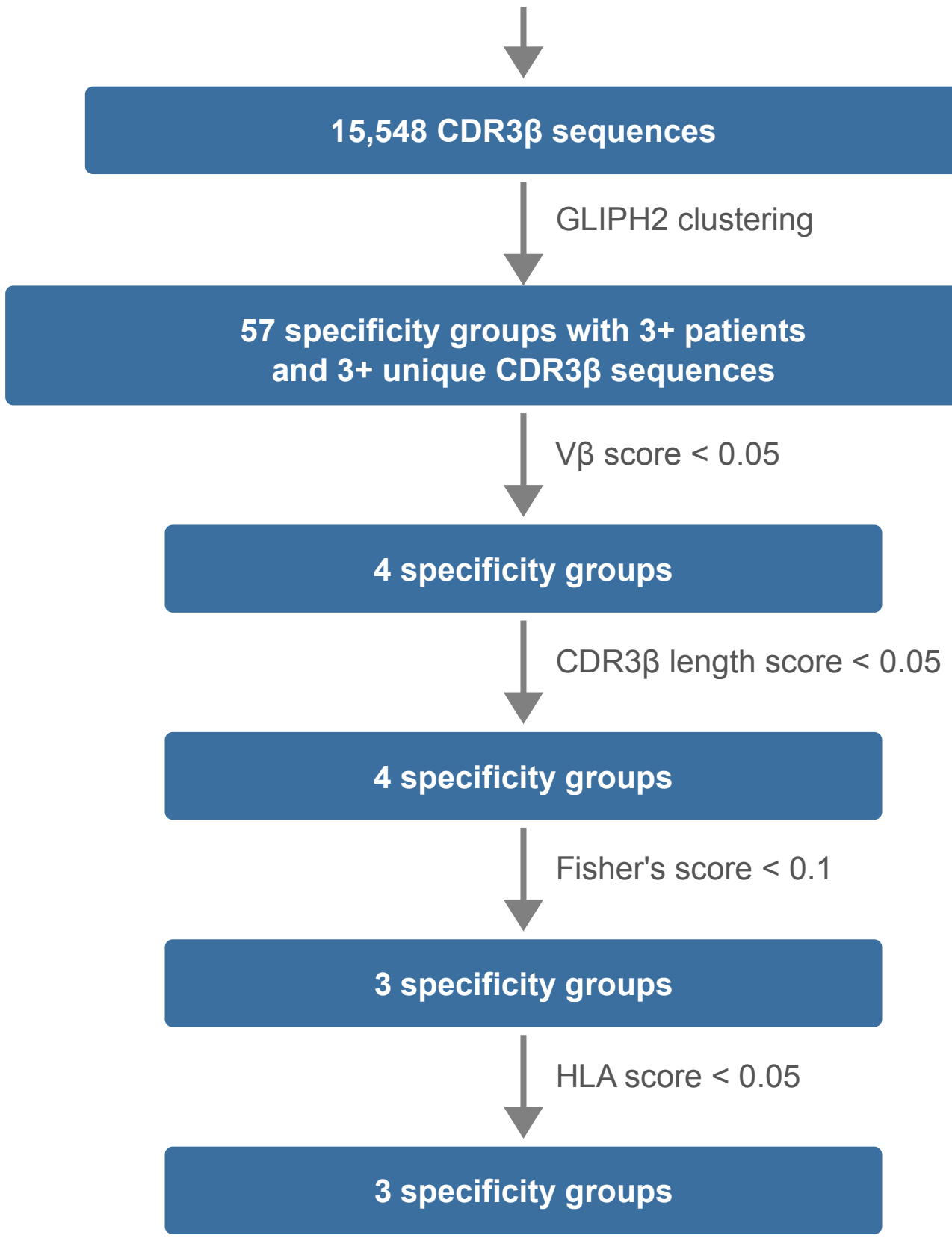

Supplementary Figure 5: GLIPH2 filtering workflow of KIRs+CD8+ TCRs from our IBD cohort and the Li et al. cohort of healthy controls, autoimmune disease patients, and COVID-19 patients.

Supplementary Figure 6

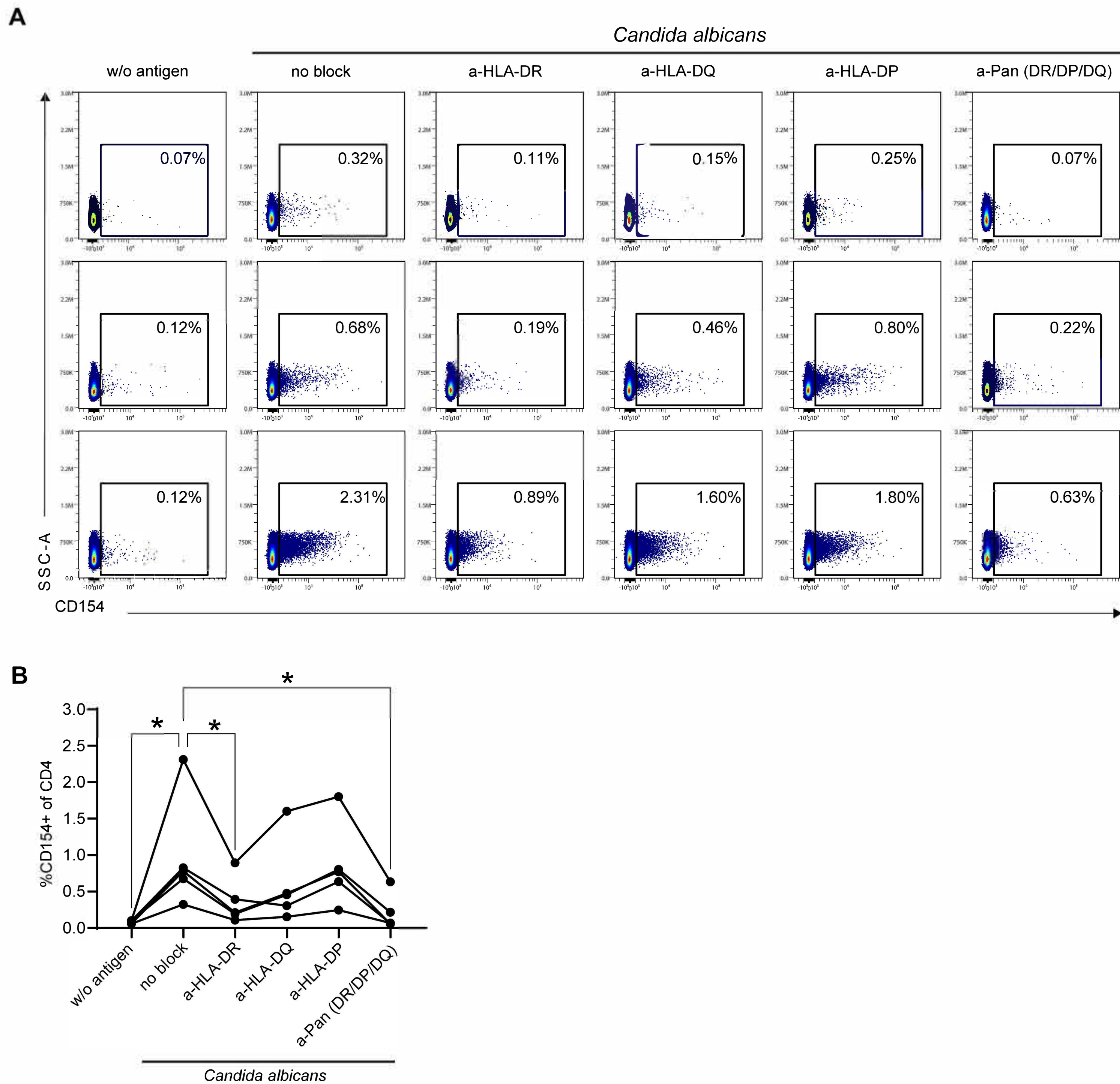

Supplementary Figure 6: Candida albicans-reactive CD4+ T cells are presented via HLA-DR.
(A) Representative flow cytometry plots showing CD154 (CD40L) upregulation on CD4+ T cells from three healthy donors following stimulation with C. albicans antigen, with or without blocking antibodies targeting HLA-DR, HLA-DQ, HLA-DP, or all three (pan-block: DR/DP/DQ). The no-antigen condition serves as a negative control. Numbers indicate the percentage of CD154+ cells within the gated population. **(B)** Summary plot of %CD154+ CD4+ T cells across all conditions for each donor. Statistical comparisons were made against the unblocked C. albicans-stimulated condition using a Friedman test with FDR correction for multiple comparisons. *$p < 0.05$.

Supplementary Table 1: HLA-DRB1 allele frequencies in the cohort, categorized by disease subtype. Significant risk alleles for IBD, as determined by Goyette et al (DOI: 10.1038/ng.3176) are bolded.

HLA Allele	Healthy (n=28)	UC (n=40)	CD (n=26)
DRB1*01:01	2 (7.1%)	2 (5.0%)	0 (0.0%)
**DRB1*01:02**	1 (3.6%)	1 (2.5%)	1 (3.8%)
**DRB1*01:03**	0 (0.0%)	2 (5.0%)	0 (0.0%)
DRB1*03:01	3 (10.7%)	4 (10.0%)	2 (7.7%)
DRB1*03:12	0 (0.0%)	0 (0.0%)	1 (3.8%)
DRB1*04:01	2 (7.1%)	2 (5.0%)	1 (3.8%)
DRB1*04:02	1 (3.6%)	0 (0.0%)	1 (3.8%)
DRB1*04:03	0 (0.0%)	1 (2.5%)	1 (3.8%)
DRB1*04:04	1 (3.6%)	0 (0.0%)	0 (0.0%)
**DRB1*04:05**	3 (10.7%)	0 (0.0%)	0 (0.0%)
DRB1*04:07	1 (3.6%)	0 (0.0%)	0 (0.0%)
DRB1*04:11	0 (0.0%)	1 (2.5%)	0 (0.0%)
**DRB1*07:01**	2 (7.1%)	2 (5.0%)	2 (7.7%)
**DRB1*08:01**	0 (0.0%)	0 (0.0%)	1 (3.8%)
DRB1*08:02	0 (0.0%)	1 (2.5%)	0 (0.0%)
**DRB1*08:03**	1 (3.6%)	0 (0.0%)	0 (0.0%)
DRB1*08:04	0 (0.0%)	0 (0.0%)	1 (3.8%)
DRB1*09:01	0 (0.0%)	0 (0.0%)	1 (3.8%)
DRB1*10:01	1 (3.6%)	0 (0.0%)	0 (0.0%)
**DRB1*11:01**	1 (3.6%)	5 (12.5%)	1 (3.8%)
DRB1*11:03	1 (3.6%)	0 (0.0%)	0 (0.0%)
**DRB1*11:04**	0 (0.0%)	1 (2.5%)	0 (0.0%)
**DRB1*12:01**	0 (0.0%)	4 (10.0%)	3 (11.5%)
**DRB1*13:01**	0 (0.0%)	3 (7.5%)	1 (3.8%)
**DRB1*13:02**	0 (0.0%)	1 (2.5%)	1 (3.8%)
**DRB1*13:05**	0 (0.0%)	1 (2.5%)	0 (0.0%)
DRB1*14:04	0 (0.0%)	1 (2.5%)	0 (0.0%)
DRB1*14:06	0 (0.0%)	1 (2.5%)	0 (0.0%)
DRB1*14:54	2 (7.1%)	1 (2.5%)	1 (3.8%)
**DRB1*15:01**	5 (17.9%)	1 (2.5%)	4 (15.4%)
**DRB1*15:02**	1 (3.6%)	4 (10.0%)	3 (11.5%)
DRB1*16:01	0 (0.0%)	1 (2.5%)	0 (0.0%)

Supplementary Table 2: (A) Ten healthy-associated and five IBD-associated TCR specificity groups (p<0.05). A two-sided p-value was obtained from Fisher's exact test (B) UC-CD enrichment comparisons, with a two-sided p-value was obtained from Fisher's exact test.

**(A) Healthy-associated GLIPH clusters**					
**GLIPH2 cluster**	**# HCs with TCRs in cluster**	**# HCs without TCRs in cluster**	**# IBD with TCRs in cluster**	**# IBD without TCRs in cluster**	**p-value**
SDS%GYE	5	9	2 (CD n=0; UC n=2)	31	0.017
PG%AGE	6	8	3 (CD n=2; UC n=1)	30	0.012
SE%KT	5	9	3 (CD n=3; UC n=0)	30	0.037
SEGGV%	5	9	3 (CD n=0; UC n=3)	30	0.037
SLSTGG%G	5	9	3 (CD n=1; UC n=2)	30	0.037
RP%E	6	8	4 (CD n=3; UC n=1)	29	0.045
SV%GGDQP	6	8	4 (CD n=2; UC n=2)	29	0.045
SVGS%NSP	7	7	5 (CD n=2; UC n=3)	28	0.024
SL%GEGG	9	5	9 (CD n=3; UC n=6)	24	0.022
SERQ%NTE	8	6	8 (CD n=4; UC n=4)	25	0.042

**(B) IBD-associated GLIPH clusters**					
**GLIPH2 cluster**	**# HCs with TCRs in cluster**	**# HCs without TCRs in cluster**	**# IBD with TCRs in cluster**	**# IBD without TCRs in cluster**	**p-value**
SESG%GQP	0	14	11 (CD n=4; UC n=7)	22	0.021
RD%LYG	0	14	10 (CD n=4; UC n=6)	23	0.023
S%RGATGE	0	14	10 (CD n6; UC n=4)	23	0.023
%IGSGANV	0	14	9 (CD n=3; UC n=6)	24	0.043
S%LDGYE	0	14	9 (CD n=5; UC n=4)	24	0.043

**GLIPH2 cluster**	**UC (n/N, %)**	**CD (n/N, %)**	**HC (n/N, %)**	**UC vs. CD p-value**
SESG%GQP	7/20 (35.0%)	4/13 (30.8%)	0/14 (0%)	1
RD%LYG	6/20 (30.0%)	4/13 (30.8%)	0/14 (0%)	1
S%RGATGE	4/20 (20.0%)	6/13 (46.2%)	0/14 (0%)	0.14
%IGSGANV	6/20 (30.0%)	3/13 (23.1%)	0/14 (0%)	1
S%LDGYE	4/20 (20.0%)	5/13 (38.5%)	0/14 (0%)	0.43

Supplementary Table 3: Study participants' HLA alleles and TCRs in healthy and disease-associated TCR specificity groups. Cells marked with "–" denote that single-cell RNA sequencing was not performed on that patient; thus, cytotoxic gene expression could not be performed.

Patient	Sex	Age	Ethnicity	Medication	Biologic?	Montreal Classification	HLA-DRB1 allele 1	HLA-DRB1 allele 2	%IGSGANV (# TCRs)	S%LDGYE (# TCRs)	S%RGATGE (# TCRs)	SESG%GQP (# TCRs)	RD%LYG (# TCRs)	SDS%GYE (# TCRs)	PG%AGE (# TCRs)	SE%KT (# TCRs)	SEGGV% (# TCRs)	SLSTGG%G (# TCRs)	RP%E (# TCRs)	SV%GGDQP (# TCRs)	SVGS%NSP (# TCRs)	SL%GEGG (# TCRs)	SERQ%NTE (# TCRs)	Cytotoxic CD4+ cells?	KIR+CD8+ cells?
01_Healthy	Female	54	Unknown	None	No		DRB1*04:01	DRB1*04:01	0	0	0	0	0	1	1	1	0	0	0	0	0	0	10	–	No
05_Healthy	Female	31	Unknown	None	No		DRB1*03:01	DRB1*11:03	0	0	0	0	0	0	2	0	0	1	1	1	3	1	0	–	No
07_Healthy	Female	54	Caucasian	None	No		DRB1*03:01	DRB1*04:02	0	0	0	0	0	0	0	0	0	0	0	2	2	0	0	–	No
11_Healthy	Female	44	Caucasian	None	No		DRB1*15:01	DRB1*15:02	0	0	0	0	0	4	0	0	3	8	1	0	0	5	5	–	No
14_Healthy	Male	32	Caucasian	None	No		DRB1*01:02	DRB1*11:01	0	0	0	0	0	2	1	0	0	1	1	0	1	0	6	–	No
15_Healthy	Female	24	Caucasian	None	No		DRB1*01:01	DRB1*14:54	0	0	0	0	0	0	0	3	2	0	1	1	0	0	0	–	No
18_Healthy	Unknown	Unknown	Unknown	None	No		DRB1*14:54	DRB1*15:01	0	0	0	0	0	0	0	0	2	0	1	3	0	5	3	–	No
22_Healthy	Unknown	Unknown	Unknown	None	No		DRB1*04:05	DRB1*04:05	0	0	0	0	0	10	0	1	0	1	0	3	0	0	0	–	No
25_Healthy	Unknown	Unknown	Unknown	None	No		DRB1*07:01	DRB1*15:01	0	0	0	0	0	0	2	0	1	0	0	1	0	1	0	–	No
31_Healthy	Male	70	Hispanic/Latino	None	No		DRB1*04:07	DRB1*07:01	0	0	0	0	0	0	0	0	0	0	1	0	0	0	0	–	No
35_Healthy	Male	60	Caucasian	None	No		DRB1*10:01	DRB1*15:01	0	0	0	0	0	1	1	0	2	0	1	2	0	1	1	–	No
38_Healthy	Unknown	Unknown	Unknown	None	No		DRB1*04:05	DRB1*08:03	0	0	0	0	0	0	0	3	0	7	0	2	1	0	1	–	No
43_Healthy	Male	79	Unknown	Unknown	No		DRB1*01:01	DRB1*15:01	0	0	0	0	0	1	0	0	0	0	0	0	0	0	2	–	No
46_Healthy	Unknown	Unknown	Unknown	None	No		DRB1*03:01	DRB1*04:04	0	0	0	0	0	0	1	1	0	0	1	2	1	1	0	–	No
13_Remission_CD	Male	29	Caucasian	Remicade/Methotrexate/Infliximab	Yes	L1	DRB1*04:01	DRB1*08:01	0	0	0	1	0	0	0	0	0	0	0	0	1	0	0	–	Yes
19_Remission_CD	Female	39	Caucasian	Remicade/Mesalamine	Yes	L1	DRB1*03:01	DRB1*04:03	0	0	0	0	0	0	1	0	0	0	0	0	0	0	0	–	No
28_Remission_CD	Female	65	Unknown	Unknown	No	L1	DRB1*12:01	DRB1*15:01	0	2	0	1	0	0	0	0	0	0	0	0	0	0	1	No	No
34_Remission_CD	Male	35	South India	Remicade/Imuran	Yes	L2	DRB1*07:01	DRB1*15:02	0	0	1	0	0	1	0	0	1	0	4	1	0	0	0	No	No
39_Remission_CD	Male	46	Hispanic/Latino	Mesalamine	No	L3	DRB1*03:12	DRB1*14:54	4	0	0	1	0	0	1	0	0	0	1	0	0	0	0	–	No
02_Active_CD	Male	62	Caucasian	Mesalamine	No	L2	DRB1*01:02	DRB1*04:02	0	0	0	0	0	0	0	0	0	0	0	0	0	2	0	–	No
06_Active_CD	Female	29	Caucasian	None	No	L1	DRB1*08:04	DRB1*12:01	0	0	0	0	0	0	0	0	0	0	0	0	0	0	0	Yes	Yes
10_Active_CD	Female	29	Caucasian	Entyvio	Yes	L1	DRB1*13:02	DRB1*15:01	0	2	0	3	2	0	3	0	1	0	1	0	0	3	0	–	No
12_Active_CD	Male	32	Unknown	Azathioprine/Sulfasalazine	Yes	L1	DRB1*11:01	DRB1*13:01	1	0	1	0	2	0	0	0	1	0	0	0	0	0	1	No	No
26_Active_CD	Male	23	Filipino	Ustekinumab	Yes	L1	DRB1*15:02	DRB1*15:02	0	0	1	0	0	0	0	0	0	0	0	0	0	0	0	–	No
27_Active_CD	Male	57	Caucasian	None	No	L1	DRB1*12:01	DRB1*15:01	4	2	0	1	0	1	0	0	0	0	1	6	0	0	0	–	No
30_Active_CD	Unknown	Unknown	Unknown	Unknown	No	L2	DRB1*03:01	DRB1*07:01	0	0	2	0	0	0	0	0	0	0	0	0	0	0	0	–	No
40_Active_CD	Unknown	Unknown	Unknown	Mesalamine	No	L3	DRB1*09:01	DRB1*15:01	0	3	0	1	1	0	0	0	0	0	0	1	0	0	0	–	No
03_Remission_UC	Female	31	Unknown	Entyvio	Yes	E1	DRB1*07:01	DRB1*12:01	1	1	1	1	0	0	0	1	0	0	0	0	0	0	0	Yes	Yes
08_Remission_UC	Female	26	Hispanic/Latino	Azathioprine/Sulfasalazine	No	E1	DRB1*08:02	DRB1*15:02	0	0	1	0	0	1	0	0	0	0	2	1	0	0	0	No	No
21_Remission_UC	Female	62	Caucasian	Infliximab, Mesalamine	Yes	E1	DRB1*03:01	DRB1*14:06	0	0	0	0	0	0	1	0	0	0	0	1	1	0	0	–	No
24_Remission_UC	Unknown	Unknown	Unknown	Unknown	No	E1	DRB1*07:01	DRB1*16:01	0	0	0	0	0	0	0	0	0	0	0	0	0	0	0	–	No
29_Remission_UC	Male	25	Unknown	None	No	E1	DRB1*13:01	DRB1*15:01	0	0	0	0	2	0	0	0	0	0	0	0	0	0	0	–	No
32_Remission_UC	Female	22	Unknown	Unknown	No	E1	DRB1*04:03	DRB1*04:11	0	1	0	0	7	0	0	0	0	0	0	0	0	0	1	–	No
36_Remission_UC	Unknown	Unknown	Unknown	Unknown	No	E1	DRB1*12:01	DRB1*13:01	1	1	0	1	0	0	0	0	0	0	0	0	1	0	0	–	No
41_Remission_UC	Female	29	Asian Indian	Mesalamine	No	E1	DRB1*15:02	DRB1*15:02	0	2	0	1	1	0	0	0	0	3	1	2	0	0	2	No	No
44_Remission_UC	Male	38	Caucasian	Tofacitinib/Vedolizumab	Yes	E1	DRB1*01:03	DRB1*01:03	0	0	0	0	0	0	0	0	0	0	0	0	0	0	0	–	No
04_Active_UC	Male	27	Unknown	Remicade/Methotrexate	Yes	E3	DRB1*11:01	DRB1*15:02	1	1	6	0	3	0	0	0	0	1	2	1	0	1	2	–	No
09_Active_UC	Male	66	Caucasian	Entyvio	Yes	E3	DRB1*03:01	DRB1*11:01	0	0	1	2	0	0	0	0	0	0	0	0	0	1	0	–	No
16_Active_UC	Female	24	Caucasian	Vedolizumab	Yes	E1	DRB1*03:01	DRB1*03:01	1	0	0	0	0	0	0	0	0	0	0	1	0	0	0	–	No
17_Active_UC	Female	39	Caucasian	None	No	E3	DRB1*11:04	DRB1*13:01	0	0	0	0	3	0	0	0	0	0	0	0	0	0	0	No	No
20_Active_UC	Male	40	Caucasian	Vedolizumab	Yes	E2	DRB1*01:01	DRB1*01:01	4	0	0	0	0	0	0	0	0	0	1	1	0	0	0	–	No
23_Active_UC	Male	53	South India	Vedolizumab/Balsalazide/Mesalamine/Corticosteroids	Yes	E2	DRB1*11:01	DRB1*14:04	0	0	0	0	0	0	0	0	0	2	0	0	0	0	0	Yes	Yes
33_Active_UC	Unknown	Unknown	Unknown	Unknown	No	E3	DRB1*12:01	DRB1*14:54	1	0	0	0	0	0	0	0	0	0	0	0	0	0	0	–	No
37_Active_UC	Male	28		Vedolizumab/Mesalamine	Yes	E3	DRB1*11:01	DRB1*12:01	0	0	0	0	1	0	0	0	0	0	0	0	0	0	0	No	No
42_Active_UC	Female	22	Caucasian	Mesalamine	No	E3	DRB1*04:01	DRB1*13:05	0	1	0	0	0	0	0	1	0	0	0	0	0	0	0	No	Yes
45_Active_UC	Female	34	Caucasian	Prednisone	No	E1	DRB1*11:01	DRB1*13:02	0	0	0	0	1	0	0	0	0	0	0	0	0	0	0	Yes	No
47_Active_UC	Female	31	Unknown	Mesalamine	No	E1	DRB1*01:02	DRB1*04:01	0	0	0	0	0	0	0	0	0	0	0	0	0	0	0	–	No

Supplementary Table 4: Genes differentially enriched in GZMB+ PRF1+ cells (UMAP cluster 8) compared to all other cell types. Gene expression was measured in a subset of 12 patients with HLA-DRB1 risk alleles for IBD and TCRs in IBD-enriched GLIPH2 specificity groups. A one-sided Fisher's exact test was performed, with and without FDR correction.

Gene	Avg log2(fold-change)	P-value	FDR-adjusted p-value
NKG7	5.823416208	0	0
GZMH	5.691189426	0	0
GZMB	4.694018156	0	0
GNLY	4.498637681	0	0
ZNF683	4.337006527	0	0
GZMA	4.225422956	0	0
CCL5	3.682064226	0	0
CST7	3.506992997	0	0
CCL4	3.46753423	0	0
PRF1	3.241838997	0	0
GZMK	3.141449516	0	0
ITGAM	2.906271291	0	0
TBX21	2.312822335	0	0
TARP-refseq	2.175896099	0	0
CTSW	2.078520298	0	0
HLA.DRB3	1.850622747	0	0
CD300A	1.796401231	4.10E-240	1.71E-239
LAIR2	1.652879848	2.44E-162	7.40E-162
HLA.DPA1	1.364168084	2.27E-288	1.03E-287
APOBEC3G	1.353038978	4.74E-195	1.69E-194
HLA.DPB1	1.272591322	0	0
IL32	1.200872466	2.48E-296	1.18E-295
ITGB2	1.176156291	0	0
ITGAL	0.92443805	9.00E-220	3.46E-219
DUSP2	0.900759961	2.40E-194	8.26E-194
SELPLG	0.715104291	1.31E-111	3.37E-111
CD52	0.699783655	2.12E-222	8.49E-222
CD3G	0.627707814	6.06E-89	1.44E-88
TSPAN32	0.612900802	8.79E-46	1.57E-45
ANXA5	0.585612033	4.25E-72	8.85E-72
HLA.DRA	0.556661151	3.26E-39	5.43E-39
CD4	0.552516427	1.84E-95	4.48E-95
BIN2	0.519860687	2.91E-77	6.33E-77
CLEC2D	0.496674244	2.88E-35	4.37E-35
LAT	0.430070016	1.33E-67	2.70E-67
STAT6	0.422189206	2.51E-42	4.26E-42
ITGA4	0.411433434	6.97E-60	1.39E-59
CXCR3	0.385941736	8.29E-50	1.56E-49
LGALS1	0.362746409	5.14E-76	1.09E-75

Supplementary Table 5: CDR3β sequences within the three specificity groups obtained after GLIPH2 analysis and filtering of KIRs+CD8+ TCRs from our IBD patient cohort and an independent cohort comprising healthy controls, autoimmune disease patients, and COVID-19 patients. The V gene, J gene, patient, clonal expansion count, and HLA class-I alleles associated with every CDR3β sequence are also shown. HLA association for each GLIPH2 cluster, with the enriched allele per cluster marked with "!", was assessed by testing enrichment of shared HLA alleles among donors contributing TCRs to the cluster using Fisher's exact test with FDR correction.

CDR3β	V gene	J gene	Patient	Clonal frequency	HLA-A HLA-B HLA-C
CASSLsYEQYF	TRBV3-1/3-2	TRBJ2-7	03_Remission_UC	1	A*01:01/A*24:02<br>B*44:02/B*57:01!<br>C*05:01/C*06:02
CASSLrYEQYF	TRBV28	TRBJ2-7	55689-0031:COVID-19	1	A*24:07/A*34:01<br>B*15:01/B*15:02<br>C*01:02/C*08:01!
CASSLaYEQFF	TRBV5-1	TRBJ2-1	CeD60:CeD	1	A*01:01/A*29:02<br>B*14:02/B*57:01!<br>C*06:02/C*08:02!
CASSLgYEQFF	TRBV5-1	TRBJ2-1	BBS79:SLE	1	A*02:06/A*23:17<br>B*08:01/B*40:02<br>C*03:04/C*07:02

CDR3β	V gene	J gene	Patient	Clonal frequency	HLA-A HLA-B HLA-C
CASSfDSGGTDTQYF	TRBV7-2	TRBJ2-3	03_Remission_UC	1	A*01:01/A*24:02<br>B*44:02/B*57:01<br>C*05:01/C*06:02
CASSfDSGGTDTQYF	TRBV7-2	TRBJ2-3	T1D25:T1D	1	A*03:01/A*03:01<br>B*40:01/B*49:01!<br>C*03:04/C*07:01
CASSfDSGGTDTQYF	TRBV7-3	TRBJ2-3	OHC015:COVID-19	1	A*02:01/A*11:02<br>B*27:04/B*35:01<br>C*03:03/C*12:02
CASSsDSGGTDTQYF	TRBV7-2	TRBJ2-3	55689-0033:COVID-19	1	A*02:01/A*03:01<br>B*07:02/B*49:01!<br>C*07:01/C*07:02
					A*11:--/A*33:--

Supplementary Table 5: CDR3β sequences within the three specificity groups obtained after GLIPH2 analysis and filtering of KIRs+CD8+ TCRs from our IBD patient cohort and an independent cohort comprising healthy controls, autoimmune disease patients, and COVID-19 patients. The V gene, J gene, patient, clonal expansion count, and HLA class-I alleles associated with every CDR3β sequence are also shown. HLA association for each GLIPH2 cluster, with the enriched allele per cluster marked with "!", was assessed by testing enrichment of shared HLA alleles among donors contributing TCRs to the cluster using Fisher's exact test with FDR correction.

CASSlDSGGTDTQYF	TRBV7-2	TRBJ2-3	23_Active_UC	1	B*51:01/B*52:01<br>C*12:02/C*14:0
CASSlDSGGTDTQYF	TRBV7-2	TRBJ2-3	55689-0033:COVID-19	1	A*02:01/A*03:01<br>B*07:02/B*49:01!<br>C*07:01/C*07:02
CASSpDSGGTDTQYF	TRBV7-3	TRBJ2-3	OHC015:COVID-19	1	A*02:01/A*11:02<br>B*27:04/B*35:01<br>C*03:03/C*12:02

CDR3β	V gene	J gene	Patient	Clonal frequency	HLA-A<br>HLA-B<br>HLA-C
CASSlGTESNQPQHF	TRBV7-2	TRBJ1-5	03_Remission_UC	2	A*01:01/A*24:02<br>B*44:02!/B*57:01<br>C*05:01!/C*06:02
CASSlGTESNQPQHF	TRBV7-2	TRBJ1-5	13_Remission_CD	1	A*03:01/A*2:01<br>B*35:01/B*44:02!<br>C*03:03/C*05:01!
CASSgGTESNQPQHF	TRBV7-2	TRBJ1-5	03_Remission_UC	1	A*01:01/A*24:02<br>B*44:02!/B*57:01<br>C*05:01!/C*06:02
CASSgGTESNQPQHF	TRBV7-2	TRBJ1-5	13_Remission_CD	1	A*01:01/A*24:02<br>B*35:01/B*44:02!<br>C*03:03/C*05:01!
CASStGTESNQPQHF	TRBV7-2	TRBJ1-5	03_Remission_UC	1	A*03:01/A*2:01<br>B*44:02!/B*57:01<br>C*05:01!/C*06:02
CASStGTESNQPQHF	TRBV7-2	TRBJ1-5	13_Remission_CD	1	A*01:01/A*24:02<br>B*35:01/B*44:02!<br>C*03:03/C*05:01!
CASSpGTESNQPQHF	TRBV7-2	TRBJ1-5	03_Remission_UC	1	A*03:01/A*2:01<br>B*44:02!/B*57:01<br>C*05:01!/C*06:02

Supplementary Table 5: CDR3β sequences within the three specificity groups obtained after GLIPH2 analysis and filtering of KIRs+CD8+ TCRs from our IBD patient cohort and an independent cohort comprising healthy controls, autoimmune disease patients, and COVID-19 patients. The V gene, J gene, patient, clonal expansion count, and HLA class-I alleles associated with every CDR3β sequence are also shown. HLA association for each GLIPH2 cluster, with the enriched allele per cluster marked with "!", was assessed by testing enrichment of shared HLA alleles among donors contributing TCRs to the cluster using Fisher's exact test with FDR correction.

CASSpGTESNQPQHF	TRBV7-2	TRBJ1-5	13_Remission_CD	1	A*01:01/A*24:02<br>B*35:01/B*44:02!<br>C*03:03/C*05:01!
CASSfGTESNQPQHF	TRBV7-2	TRBJ1-5	03_Remission_UC	1	A*03:01/A*2:01<br>B*44:02!/B*57:01<br>C*05:01!/C*06:02
CASSfGTESNQPQHF	TRBV7-2	TRBJ1-5	13_Remission_CD	1	A*01:01/A*24:02<br>B*35:01/B*44:02!<br>C*03:03/C*05:01!
CASSsGTESNQPQHF	TRBV7-2	TRBJ1-5	03_Remission_UC	2	A*03:01/A*2:01<br>B*44:02!/B*57:01<br>C*05:01!/C*06:02
CASSsGTESNQPQHF	TRBV7-2	TRBJ1-5	13_Remission_CD	1	A*03:01/A*2:01<br>B*35:01/B*44:02!<br>C*03:03/C*05:01!
CASSsGTESNQPQHF	TRBV7-2	TRBJ1-5	23_Active_UC	2	A*01:01/A*24:02<br>B*51:01/B*52:01<br>C*12:02/C*14:0
